# Inhibition of the Pim1 Oncogene Results in Diminished Visual Function

**DOI:** 10.1371/journal.pone.0052177

**Published:** 2012-12-26

**Authors:** Jun Yin, Lisa Shine, Francis Raycroft, Sudhakar Deeti, Alison Reynolds, Kristin M. Ackerman, Antonino Glaviano, Sean O'Farrell, Olivia O'Leary, Claire Kilty, Ciaran Kennedy, Sarah McLoughlin, Megan Rice, Eileen Russell, Desmond G. Higgins, David R. Hyde, Breandan N. Kennedy

**Affiliations:** 1 UCD School of Medicine and Medical Science, UCD Conway Institute, University College Dublin, Dublin, Ireland; 2 UCD School of Biomolecular and Biomedical Science, UCD Conway Institute, University College Dublin, Dublin, Ireland; 3 Department of Biological Sciences and the Center for Zebrafish Research, University of Notre Dame, Notre Dame, Indiana, United States of America; Wayne State University School of Medicine, United States of America

## Abstract

Our objective was to profile genetic pathways whose differential expression correlates with maturation of visual function in zebrafish. Bioinformatic analysis of transcriptomic data revealed Jak-Stat signalling as the pathway most enriched in the eye, as visual function develops. Real-time PCR, western blotting, immunohistochemistry and in situ hybridization data confirm that multiple Jak-Stat pathway genes are up-regulated in the zebrafish eye between 3–5 days post-fertilisation, times associated with significant maturation of vision. One of the most up-regulated Jak-Stat genes is the proto-oncogene Pim1 kinase, previously associated with haematological malignancies and cancer. Loss of function experiments using Pim1 morpholinos or Pim1 inhibitors result in significant diminishment of visual behaviour and function. In summary, we have identified that enhanced expression of Jak-Stat pathway genes correlates with maturation of visual function and that the Pim1 oncogene is required for normal visual function.

## Introduction

Our objective was to investigate the molecular genetics regulating maturation of visual function in vertebrates. Development of the zebrafish visual system is rapid with morphogenesis of the optic vesicles beginning at ∼10 hours post-fertilisation (hpf) [1]. Rapid proliferation and progressive lamination follows. By ∼72 hpf, most retinal cell types are distinguishable, and lamination of the retina does not significantly change from 3–5 days post-fertilisation (dpf). However, progression from a morphologically developed eye, to an eye with robust visual function occurs between 3–5 dpf [2], [3].

A light-evoked locomotor response is detected in zebrafish at ∼68 hpf [3]. This startle response likely recapitulates an escape response invoked by the shadow of an approaching predator [4]. Initially known as the shadow-induced startle response, it was first assessed by placing larvae in a petri dish, extinguishing a light source for 1 second and observing whether larvae moved in response. The related visual motor response (VMR) is assessed using an automated system which uses an infrared camera to quantify the movement of larvae in response to lights turned on or off [4]. Another visual response, the optokinetic response (OKR) represents the ability of zebrafish to detect contrasting patterns and is detected from 73 hpf [3], [5]. The initial OKR is slow and sporadic, but improves so that by 96 hpf, larvae track the drum analogous to adult fish and by 5 dpf, the response is adult-like [6]. The first electrical responses from the retina have been detected as early as 72 hpf [7]. These responses are also small in amplitude, requiring high intensity stimuli. Zebrafish electroretinograms (ERG) are typically recorded from 5 dpf larvae in which responses are more robust [8].

Here, we avail of Affymetrix GeneChip technology to globally profile genes with significant differential expression in the zebrafish eye between 3–5 dpf, as visual function matures. Interestingly, significantly enhanced expression of Jak-Stat signalling genes, a pathway typically associated with interferon and cytokine signalling, correlates with maturation of visual function [9]. Pim1–2 kinases, proto-oncogenes and downstream components of Jak-Stat signalling, unexpectedly displayed differential expression in the developing eye [10]. Pharmacological and genetic inhibition of Pim1 kinase results in a specific disruption of visual behaviour and retinal function. These results highlight a novel role for the Pim1 kinase in visual function.

## Materials and Methods

### Microarray experiment

Zebrafish were maintained according to standard procedures on a 14 h light/10 h dark cycle at 28°C. Embryos were obtained by natural spawning and developmental stages established by time and morphological criteria. Microarray experiments were performed as previously described [11]. Eyes were dissected from 3, 4 and 5 days post fertilization (dpf) zebrafish larvae. Total RNA was extracted and labeled using a two-cycle target labelling protocol (Affymetrix, Santa Clara, USA) and hybridised with Affymetrix Zebrafish Genome Arrays. Three biological replicates per time point were used with equal amounts of RNA. The 3, 4 and 5 dpf eyes microarray data set was deposited in GEO with accession ID GSE19320. All experimental protocols were approved by the UCD Animal Research Ethics Committee, and the University of Notre Dame Animal Care and Use Committee.

### Zebrafish genome reannotation and probe remapping

Gene annotation was based on the zebrafish genome version 9 (Zv9) and integrating gene transcript collections from multiple genome annotation databases [11]. Transcript data from the RefSeq, GenBank and Ensembl databases were downloaded from the UCSC genome browser [12]. Transcripts were clustered into genes from overlapping coding exons. A customized probe remapping was performed as previously described [11]. In order to take advantage of the human genome annotation, human-zebrafish homology data were downloaded from Ensembl [13], BioMart [14], ZFIN [15], and NCBI HomoloGene [16]. These homology databases were combined with the zebrafish genome annotation databases. Where no functional annotation for a transcript could be found, cDNA sequences were searched against the NCBI refseq_protein database using blastx [17]. The highest scoring human homologs were identified with at least 30% identity to the query sequence over at least 30% sequence length. Human KEGG pathway [18] and Gene Ontology [19] annotations were combined with zebrafish annotations for gene set analysis. Human retinal disease information was downloaded from RETNET [20].

### Microarray data analysis

The Bioconductor package, *gcrma*, was used to normalize and summarize microarrays signal intensities [21]. Probe sets detected at low signal were removed, with maximal log transformed signal intensity <6 in all samples. The Bioconductor package, *limma*, was used to select differentially expressed genes [22]. P-values from an eBayes model-based t-test were adjusted using Benjamini & Hochberg's method [23]. The threshold for differentially expressed genes was set as adjusted p-value <0.05 and fold change ≥1.5 or ≤0.67. For genes with multiple probe sets, a revised Splicing Index is calculated [11]. If the Splicing Index is ≤1 and ≥−1, the probe set expressions were averaged to calculate gene level expression. Otherwise, the probe set expressions are used separately to predict alternative splicing patterns. Fisher's Exact Test was used to indicate the significance of enriched Gene Ontology and KEGG pathway.

### Real-time PCR Validation

Real-time PCR was performed as previously described [11]. Eyes were dissected from 3 and 5 dpf zebrafish larvae and total RNA was extracted. Three biological replicates were used for both time points. cDNA was synthesized with random hexamers using the Superscript III First-Strand Synthesis System (Invitrogen, UK). Real-time PCR was performed using the ABI 7900HT Sequence Detection System. Primers were designed using Primer-BLAST (http://www.ncbi.nlm.nih.gov/tools/primer-blast/) and synthesised by Eurofins MWG Operon (Germany). The primers for different genes are listed in Table S1. 18 s rRNA primers were used as control. Taqman probes were used as the reporter in the 18 s control samples and SYBR Green was the reporter in all other reactions. Real-time data were normalized according to 18 s rRNA.

### Histological Analysis

Whole larvae were fixed overnight in a solution of 4% paraformaldehyde and 2.5% gluteraldehyde diluted in 0.1 M Sorenson phosphate buffer (pH 7.3) at room temperature. Samples were then post-fixed in 1% osmium tetroxide in 0.1 M Sorenson phosphate buffer for 1 hour at room temperature, dehydrated in ascending concentrations of ethanol to 100% and embedded in epon resin according to standard methods. Semi-thin (1 µm) sections were cut using a glass knife and a Reichert-Jung Ultracut E microtome and visualised by light microscopy using a Nikon E80i transmission microscope

### Immunoblot Analysis

Immunoblots were performed similar to previously described (Kassen et al., 2007). Protein was harvested from ∼30 larvae, homogenized in 15 µl of extraction buffer (1× PBS/10% Glycerol/1% Triton X-100/5 mM KPO_4_/0.05 mM EDTA/1× Complete Protease Inhibitor Cocktail Tablet (Roche; Indianapolis, IN) and a tyrosine and serine/threonine phosphatase inhibitor cocktail mix (Phosphatase Inhibitor Cocktails 2 and 3, Sigma; St. Louis, MO) and stored at −20°C. After SDS-PAGE, proteins were electrotransferred to a PVDF H-Bond membrane (Amersham; Pis-cataway, NJ) and blocked in 1× PBS/0.1% Tween-20/5% non-fat dry milk overnight at 4°C. The membrane was incubated with immunopurified anti-Stat3 polyclonal antisera (1∶5,000) [24], anti-Socs1 polyclonal antisera (1∶5,000), anti-Socs3a polyclonal antisera (1∶2,000) or an anti-actin monoclonal antibody (1∶10,000, Calbiochem; San Diego, CA) overnight at 4°C in blocking buffer. The membranes were washed in 1× PBS/0.1% Tween-20 (3×10 min), and incubated for 1 hr at room temperature with either an anti-rabbit or anti-mouse HRP-conjugated secondary antibody (1∶10,000, Amersham). The membranes were washed in 1× PBS/0.1% Tween-20 (3×10 min) and the secondary antibodies were detected with the ECL-Plus system (Amersham) as described previously [25]. The NIH Image-J software was used to quantify band intensities on the immunoblots. For each time point, the intensity of the actin control band was normalized to the 2 dpf band. For each polyclonal antiserum, the intensity of the band at each time point was calculated relative to the actin control at the same time point and the relative amount of each protein at 2 dpf was set to 1.0. Plotted are the natural log of the mean values (n = 3) and the standard error of the means.

### Generation of Anti-Socs1 and Anti-Socs3a Polyclonal Antisera

The polyclonal Stat3 antisera used in this study was previously described [24]. To generate polyclonal antisera against the Socs1 (NP_001003467.1), and Socs3a (NP_956244.1) proteins, an amino terminal segment of zebrafish Socs1 corresponding to amino acids 1–67 and an interior segment of zebrafish Socs3a corresponding to amino acids 13–50 were expressed as bacterial fusion proteins using the pET32a vector (Novagen, San Diego, CA). The fusion proteins were purified using S-protein agarose and used to immunize rabbits (Proteintech Group, Chicago, IL). The same fusion proteins were coupled to separate gel matrix columns according to the manufacturer protocol (AminoLink Coupling Gel; Pierce Biotechnology, Rockford, IL) and the anti-Socs1 and anti-Socs3a polyclonal rabbit antisera immunopurified over these columns.

### Immunohistochemistry

Wild-type zebrafish larvae were fixed in 4% paraformaldehyde in 5% sucrose/1× PBS, washed in 5% sucrose/1× PBS at room temperature, cryoprotected in 30% sucrose/1× PBS overnight at 4°C and embedded in Tissue Freezing Medium or OCT. 10–12 µm sections were cut and thaw-mounted onto charged slides. The sections were rehydrated using PBS and blocked for 1 hr using 2% (vol/vol) normal goat serum, 1% bovine serum albumin and 0.1% Triton X-100 or 2% normal goat serum/0.2% Triton X-100/1% DMSO, in PBS. Sections were incubated overnight at 4°C with the primary antibody diluted in blocking buffer (anti-Stat3 1∶200, anti-Socs1 1∶50, anti-Socs3a 1∶50 or anti-Pim1 (K0267; Sigma-Aldrich) 1∶200) Slides were washed in PBS before being incubated with a 1∶200 dilution of a Cy3-conjugated goat anti-rabbit antibody in 1% Triton X-100/PBS or a AF594-conjugated goat anti-rabbit IgG secondary antibody (2 mg/ml, Molecular Probes; Eugene, OR) diluted 1∶500 in blocking buffer. After washing with PBS the slides were washed with PBS and mounted in Aqua Poly/Mount (Polysciences Inc.) or ProLong Gold (Invitrogen) Sections were imaged using a fluorescent microscope (Axioplan 2; Carl Zeiss Meditec, Inc. or a Leica TCS SP2 laser scanning confocal microscope).

### In situ hybridization

Total RNA was isolated from zebrafish embryos at 5 dpf using Trizol (Invitrogen) and reverse transcribed using random primers with the Superscript III Preamplification System (Invitrogen). The Socs1, Socs3a and Stat3 cDNAs were amplified using Platinum Taq (Invitrogen), and Pim1 cDNA was amplified using Crimson taq (New England Biolabs) with primers listed in Table S1, using an annealing temperature of 60°C. PCR products were gel purified (QIAquick Gel Extraction, Qiagen). Socs1, Socs3a and Stat3 were cloned into pCR II-TOPO. Pim1 was cloned into pGEM-T Easy Vector. Plasmids were sequenced to confirm the identity of the cDNAs. The Socs1, Socs3a and Stat3 cDNA containing plasmids were linearized with either HindIII or NotI and precipitated, in vitro transcribed into antisense and sense digoxigenin (DIG)-labeled RNA probes (Roche DIG RNA Labeling Kit SP6/T7) with either T7 or SP6 RNA polymerase. Pim1 containing plasmids were linearized with either SacI or NcoI, and in vitro transcribed into antisense and sense DIG-labeled RNA probes as above. The in vitro transcription reactions were terminated by adding 0.2 M ethylenediaminetetraacetic acid (EDTA) and the riboprobes were precipitated using ammonium acetate and 100% ethanol. The quality of the in vitro transcribed RNA was confirmed by electrophoresis through a 1% agarose formaldehyde gel. Embryos were fixed overnight at 4°C in 4% paraformaldehyde (PFA) and in situ hybridization was performed on whole embryos as previously described [26]. After in situ hybridization, the embryos were re-fixed overnight at 4°C in 4% PFA, cryopreserved, and sectioned at 12 µm.

### Morpholino knockdown

Morpholino oligonucleotides were designed by Gene Tools (Gene Tools LLC, Philomath, OR) and targeted the *pim1* exon2-intron2 splice junction (5′ TCCTCCATTGAGGGAACCTACCGGC), the *pim1* exon4-intron4 splice junction (5′ GGTCATGCAAATGGCTCTTACCGTC), the *stat3* translation blocking (5′ CAGATAAATCGTCCTCCACGGAAAC), the *socs3a* translation blocking (5′ TACACACCAAACCCTGAGCTGCCGG), the *socs1* translation blocking (5′ TGCGCCACCATCCTACAGGAAAGAC), or the standard control morpholino (designed by GeneTools as not being complementary to any known sequence in the zebrafish genome). Morpholino oligonucleotides were resuspended in Danieau buffer (58 mM NaCl, 0.7 mM KCl,0.4 mM MgSO_4_, 0.6 mM Ca(NO_3_)_2_, 5.0 mM HEPES pH 7.6) and injected into wild type, 1–2 cell zebrafish embryos with phenol red tracer dye. The *stat3*, *socs3a* and *socs1* 5-base mismatch, and standard control morpholinos were injected at a final concentration of 0.25 mM. The *pim1* morpholino and standard control were injected at final concentration of 0.025 mM.

### Sequence and structure analysis of the pim gene family

Zebrafish Pim protein sequences from RefSeq database (Pim1 NP_001070859, Pim2 NP_571614 and Pim3 NP_001030150) were aligned with Pim protein sequences from other species using ClustalW [27]. The neighbour joining trees with bootstrapping were constructed using Seaview [28]. The 3-D structure of zebrafish Pim1 was predicted using the Swiss-Model alignment mode [29]. The modeling template was the human PIM1 crystal structure 3BGP from Protein Data Bank [30] and the accuracy of the predictions were indicated using Qmean values [31]. Drug docking was predicted using SwissDock with default settings [32]. The top-ranked binding model was used to infer the drug docking site. The 3-D structure of the interaction model was analyzed using Swiss-Pdbviewer [33].

### Zebrafish Drug Treatment and Functional Assay

For drug treatments with Pim1 inhibitor 2 (Tocris, USA) and Pim1 inhibitor II (EMD Millipore, USA), larvae were placed in embryo medium and incubated with drug dissolved in 0.1% or 1% DMSO at 28°C on a 14 h light/10 h dark cycle. For assessment of visual behaviour using OKR, larvae were placed in a petri dish containing embryo medium/9% methylcellulose [5]. The petri dish was placed inside a drum containing alternating black and white stripes (18° per stripe, contrast 99%) rotating at a speed of 16 rpm. The drum was rotated for 30 seconds clockwise then 30 seconds counter clockwise and the number of eye saccades counted.

The visual motor response (VMR) behavior was recorded using a Zebrabox (Viewpoint, France) infrared video tracking system. Individual larvae were placed in single wells of a 96 well plate. The assay protocol consisted of 30 min settling, followed by four 20 min periods of light ON and OFF. Assay parameters were set to detection sensitivity 10, burst 25, freeze 3 and the activity of individual larvae was integrated into 1 second bins. Peak activities were averaged from the duplicate on and off responses, respectively. In order to investigate the reversibility of the drug treatment, zebrafish larvae were treated with Pim1 inhibitor II for 1 hour at 5 dpf, the VMR recorded, then the drug was washed off using embryo medium, and after 7–8 hours settling and the VMR was recorded again.

A non-visual behavior, the touch response (TR) was analysed by touching larvae with a needle and scoring the locomotor response. To measure eye size, zebrafish larvae were immobilised in embryo medium/9% methylcellulose imaged using a brightfield microscope (Olympus SZX16 stereo zoom microscope) and eye diameter measured using Cell^F^ software (Olympus).

### Electroretinography

Zebrafish larvae from the Tü strain were treated at 3 dpf with 100 µM pim 1 inhibitor 2 dissolved in embryo medium. Control larvae were raised from 3–5 dpf in an equivalent amount of DMSO (0.1%). At 5 dpf, larvae were washed in embryo medium to remove the drug/DMSO. Electroretinography was performed on control (n = 5) and Pim1 inhibitor 2 treated (n = 15) fish following established methods [34] with the following modifications: three flash intensities were used (−2.0 log, −1.0 log and 0) and flash duration was 20 ms. Raw data from the sample groups were compared using an independent 2-sample t-test with unequal variances.

## Results

### Maturation of Visual Function in Zebrafish

**Figure 1:**
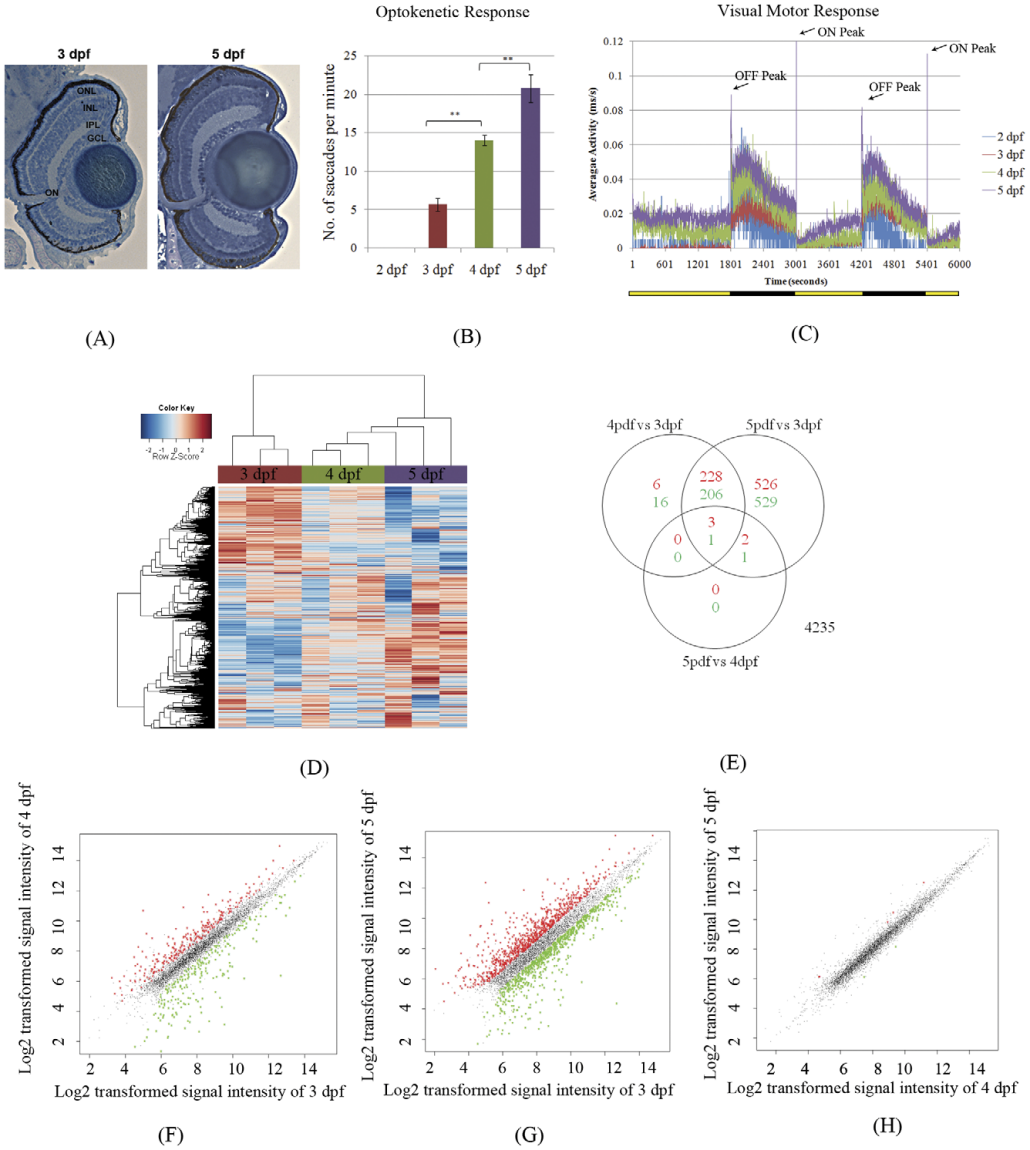
Maturation of visual function and correlations to gene expression in larval zebrafish. (A) The morphology of the zebrafish retina shows no significant changes from 3–5 dpf. However, the OKR (B) and the VMR (C) show significant increases in the number of eye saccades or the amplitude of locomotor responses to light changes from 3–5 dpf. Lights on is shown as a yellow bar below the diagram, and lights off is shown as a black bar. (D–H) Overview of microarray results of eye gene expression for 3, 4 and 5 dpf zebrafish eyes. (D) Hierarchical clustering of gene expression was based on normalized signals (z-scores) using the correlation similarity metric and average linkage clustering. (E) Venn diagram showing the number of up-regulated and down-regulated transcript-level probe sets between pairwise comparisons using the *limma* Bayesian model based t-test. (F–H) Log2 transformed signal intensities of probe sets are depicted as dots, with up-regulated probe sets in red and down-regulated sets in green.

From 3–5 dpf, the gross morphology of the zebrafish retina changes little except in size (Figure 1A), however visual function matures in this timeframe. The development of visual function in zebrafish larvae was analysed by both optokinetic response (OKR) and visual motor response (VMR) assays [4], [5]. The OKR is a visual behaviour assay in which the number of saccadic responses of the eye to rotating black and white stripes is counted. At 2 days post-fertilisation (dpf), no larvae exhibit saccadic responses (Figure 1B). At 3 dpf, ∼90% of the larvae exhibit saccadic responses; but the average response is only ∼5 saccades per minute. However, by 5 dpf, all larvae respond robustly to the rotating stripes with an average of ∼20 saccades per minute. The VMR reflects locomotor responses to changes in lighting, typified by a rapid burst of locomotor activity immediately upon turning lights ON or OFF. Similar to the OKR, the amplitude of the VMR to both lights ON and OFF significantly increases from 2–5 dpf (Figure 1C). The VMR is a readout of visual function as zebrafish larvae without eyes have significantly reduced peak responses ([4] and McLoughlin et al unpublished). In summary, visual behaviour assays of zebrafish larvae demonstrate a significant maturation of visual behaviour from 2–5 dpf.

### Transcriptomic and Bioinformatic Analysis

To identify genes whose ocular expression levels correlate with enhanced visual function, we profiled global gene expression in zebrafish eyes at 3, 4 and 5 dpf using the Affymetrix GeneChip platform. Previously, we reported a significant improvement in the accuracy of interpreting microarray datasets after integrating transcripts from multiple databases [11]. In total, 81,749 transcripts from Ensembl, GenBank and ZFIN were clustered into 29,447 genes with overlapping coding exons. To improve the specificity of probe mapping, we aligned the Affymetrix probes to zebrafish genome (Zv9). After problematic probes were filtered, the remaining 142,712 probes were clustered into 11,460 transcript level probe sets. These represent 8,901 genes out of the 29,447 genes defined within the zebrafish genome.

### Identification of genes differentially expressed in 3–5 dpf eyes

Genes that are differentially expressed between 3 and 5 dpf eyes are candidate regulators of visual function. Prior to statistical analysis of the microarray data, a signal filter was applied to remove lowly expressed genes leaving 6,839 probe sets. The microarray data sets were normalized and summarized using the Bioconductor package *gcrma* [21], and differentially expressed genes were selected with *limma*, using the eBayes model based t-test [22] (Figure 1D–H). Hierarchical clustering of the global gene expression data shows that the 4 and 5 dpf eye transcriptomes cluster together. The largest cohort of differentially expressed genes occurs between the 5 and 3 dpf eyes with 759 probe sets up-regulated, and 737 probe sets down-regulated. The number of differentially expressed genes between 4 and 3 dpf was much smaller and mostly included in the 5 versus 3 dpf list. Therefore, genes differentially expressed between 5 versus 3 dpf were further investigated. The top 50 differentially expressed genes are shown in Table 1 and categorized by biological process using gene ontology annotation. Many of the top up-regulated genes at 5 dpf are related to signal transduction or are known targets of signalling pathways. The dual specificity phosphatase 5, *dusp5*, is a negative regulator of interleukin-2 and MAPK signalling pathways [35]. Bcl2-related ovarian killer b, *bokb*, and Bcl2 interacting protein 3, *bnip3*, relate to Bcl signalling. Bcl2 is a downstream target of the Jak-Stat signalling pathway [36]. At 3 dpf, many of the significantly down-regulated genes are related with muscle and muscle contraction. Genes encoding tropomyosin (*tpm3*), myosin (*myl1*), actin (*acta1a*), troponin (*tnnt3*) and collagen (*col2a1a*, *col9a2*, *col9a3* and *col11a1*) show significantly higher expression at 3 dpf. The top 20 unknown genes (Table 2) only show homology to human proteins and represent novel ESTs expressed during late development of the eye.

**Table 1:** Top 50 differentially expressed genes between 5 versus 3 dpf.

Gene Symbol	Description	Log2(Fold Change)	Q-value	Chromosome location	Adhesion	Cell cycle	Death	Development	Muscle development and system process	Regulation	Response to stress/stimulus	Signal transduction	Transcription	Transport	Vision and light stimulus
*cryga*	crystallin, gamma A	7.390	0.0000	chr9:23050725-23051699											
*bokb*	BCL2-related ovarian killer b	3.670	0.0001	chr2:22097899-22108106			+			+					
*dusp5*	dual specificity phosphatase 5	3.010	0.0002	chr22:32600889-32612552				+		+	+	+			
*npas4*	bHLH-PAS type transcription factor NXF;neuronal PAS domain protein 4	2.940	0.0001	chr14:31506584-31512448						+	+	+	+		
*krt17*	keratin 17	2.340	0.0001	chr22:11866189-11869262				+							
*mylipa*	myosin regulatory light chain interacting protein a	2.330	0.0002	chr19:26826400-26864319				+		+					
*bnip3*	BCL2/adenovirus E1B 19 kDa interacting protein 3	2.310	0.0002	chr17:20061572-20066455						+					
*per2*	period homolog 2 (Drosophila)	2.280	0.0000	chr2:48423773-48489628						+	+	+			+
*npy*	neuropeptide Y	2.230	0.0002	chr19:20594043-20596307						+	+	+		+	
*vps37a*	vacuolar protein sorting 37A	2.160	0.0002	chr1:15204974-15222186										+	
*ppp1r3cb*	protein phosphatase 1, regulatory (inhibitor) subunit 3Cb	1.910	0.0001	chr12:17355562-17359901						+					
*fnip1*	folliculin interacting protein 1	1.890	0.0001	chr21:43649629-43693555											
*btg2*	B-cell translocation gene 2	1.870	0.0002	chr22:754597-757144				+		+	+				
*tmem38b*	transmembrane protein 38B	1.760	0.0001	chr21:408193-432534										+	
*cic*	capicua homolog (Drosophila)	1.690	0.0001	chr19:6708080-6751038											
*tspan7*	tetraspanin 7	−1.470	0.0002	chr14:43002970-43012025						+	+	+			
*sec11a*	SEC11 homolog A (S. cerevisiae)	−1.490	0.0002	chr18:26732450-26742083						+					
*cd248*	CD248 molecule,endosialin	−1.680	0.0001	chr7:19379515-19382595											
*slc43a1a*	solute carrier family 43, member 1a	−1.700	0.0002	chr14:23079820-23101389				+						+	
*csgalnact1*	chondroitin sulfate N-acetylgalactosaminyltransferase 1	−1.830	0.0001	chr10:7319768-7382081											
*angptl7*	angiopoietin-like 7	−1.890	0.0001	chr8:48371745-48375260						+	+	+			
*tubb5*	tubulin, beta 5	−1.900	0.0001	chr16:11935792-11944618		+		+		+	+				
*plod1a*	procollagen-lysine 1, 2-oxoglutarate 5-dioxygenase 1a	−1.940	0.0001	chr8:50009081-50055886				+			+				
*mmp2*	matrix metalloproteinase 2	−2.000	0.0001	chr7:36864730-36887592				+							
*manf*	mesencephalic astrocyte-derived neurotrophic factor	−2.010	0.0001	chr22:35034584-35041263							+				
*smarcad1*	SWI/SNF-related, matrix-associated actin-dependent regulator of chromatin, subfamily a, containing DEAD/H box 1	−2.010	0.0001	chr8:30484009-30502207						+					
*calrl*	calreticulin like	−2.030	0.0002	chr2:55840094-55855004											
*postn*	periostin, osteoblast specific factor	−2.070	0.0001	chr15:33133895-33162913	+			+	+						
*col11a1*	collagen, type XI, alpha 1	−2.400	0.0001	chr24:29686546-29815838	+						+				+
*col9a3*	collagen, type IX, alpha 3	−2.500	0.0001	chr23:511310-541067				+			+				
*col9a2*	procollagen, type IX, alpha 2	−2.510	0.0000	chr19:39970826-40017220				+			+				
*fkbp9*	FK506 binding protein 9	−2.780	0.0000	chr19:44193712-44210392											
*tnnt2c*	troponin T1, skeletal, slow	−2.790	0.0001	chr4:11096482-11098385											
*lect1*	leukocyte cell derived chemotaxin 1	−3.070	0.0001	chr9:55750086-55771208				+		+					
*svopl*	SVOP-like	−3.070	0.0001	chr4:8831282-8843368										+	
*col2a1a*	collagen type II, alpha-1a	−3.100	0.0001	chr8:21733311-21757616	+			+		+	+				+
*spon2b*	spondin 2b, extracellular matrix protein	−3.240	0.0000	chr14:15209327-15214896	+			+			+				
*aspn*	asporin (LRR class 1)	−3.250	0.0000	chr22:10590115-10597661				+		+					
*fkbp10*	FK506 binding protein 10	−3.420	0.0001	chr12:15014462-15064192											
*hapln1a*	hyaluronan and proteoglycan link protein 1a	−3.470	0.0000	chr5:48101733-48116816	+										
*agr2*	anterior gradient homolog 2 (Xenopuslaevis)	−3.590	0.0001	chr19:31646945-31649496						+				+	
*mfi2*	antigen p97 (melanoma associated)	−3.970	0.0000	chr6:29805821-29824526						+				+	
*tnnt3a*	troponin T3a, skeletal, fast	−4.150	0.0000	chr25:32248470-32261728					+	+					
*gsc*	goosecoid	−4.230	0.0001	chr17:19173173-19175672				+	+	+			+		
*tpm3* a	tropomyosin 3	−4.440	0.0001	chr19:11477764-11521373					+	+					
*hapln1b*		−4.650	0.0000	chr10:44748006-44768991	+			+							
*acta1a*	actin, alpha 1a, skeletal muscle	−4.840	0.0000	chr1:54512581-54520512				+	+	+	+				
*myl1*	myosin, light chain 1, alkali; skeletal, fast	−5.130	0.0000	chr9:40046541-40057692				+	+						
*hand2*	heart and neural crest derivatives expressed transcript 2	−5.410	0.0000	chr1:38602300-38604523			+	+		+			+		
*matn1* a	matrilin 1	−8.310	0.0001	chr19:45245318-45268889											

a : these differentially expressed genes were verified using real-time PCR in our previous study [11].

**Table 2:** Top 20 unknown differentially expressed genes between 5 versus 3 dpf and their human homologs identified using BLASTX.

Gene Symbol	Representative Accession ID	Chromosome Location	Log2 (Fold Change)	Q-value	Human homolog identified using BLASTX			
					RefSeq ID	Protein Symbol	Description	BLASX Evalue
LOC557783	ENSDART00000022660	chr3:32595697-32608941	3.300	0.0004	NP_068751	RIC8A	resistance to inhibitors of cholinesterase 8 homolog A (C. elegans)	4.00E-110
si:ch211-237l4.6	NM_001033091	chr10:22982732-22986656	3.150	0.0001				
c8orf4	ENSDART00000111097	chr10:20290365-20291110	3.050	0.0002	NP_064515	C8orf4	chromosome 8 open reading frame 4	1.00E-15
LOC 556873	BC083258	chr17:30528306-30532467	2.800	0.0006	NP_955378	PFN4	profilin family, member 4	2.00E-24
fam169a	ENSDART00000124807	chr21:14073809-14104257	2.670	0.0005	NP_056381	FAM169A	family with sequence similarity 169, member A	4.00E-90
zgc:113162	NM_001020714	chr21:11590062-11634648	2.600	0.0004	NP_009101	TESK2	testis-specific kinase 2	3.00E-70
LOC 556200	ENSDART00000123381	chr11:39739623-39745008	2.370	0.0003				
b8ji39_danre	ENSDART00000049885	chr7:24744813-24893012	2.020	0.0004	NP_690618	DGKD	diacylglycerol kinase, delta 130 kDa	0.00E+00
zgc:165666	NM_001099242	chr3:46422259-46441887	1.690	0.0004	NP_037531	C16orf5	chromosome 16 open reading frame 5	9.00E-75
zgc:73324	NM_200789	chr3:17752915-17759753	1.670	0.0002	NP_079423	COQ10B	coenzyme Q10 homolog B (S. cerevisiae)	2.00E-57
zgc:162945	NM_001128809	chr7:31606262-31609395	1.660	0.0009	NP_776172	HARBI1	harbinger transposase derived 1	7.00E-46
si:ch211-195b13.1	NM_001077302	chr19:25368491-25374891	1.630	0.0007	NP_005618	SGK1	serum/glucocorticoid regulated kinase 1	2.00E-173
si:dkey-177p2.6	NM_001075116	chr20:26977416-26992361	1.580	0.0010	NP_055570	SERTAD2	SERTA domain containing 2	3.00E-20
zgc:110006	NM_001020552	chr10:36077772-36132111	1.520	0.0008	NP_001093890	C21orf91	chromosome 21 open reading frame 91	7.00E-72
LOC 569602	ENSDART00000011398	chr7:21304641-21382962	1.290	0.0012	NP_005535	IRS1	insulin receptor substrate 1	6.00E-155
zgc:112372	NM_001020768	chr3:61284536-61316553	1.260	0.0013	NP_872371	FAM100B	family with sequence similarity 100, member B	8.00E-56
si:ch211-11k18.4	ENSDART00000121613	chr3:26851370-26860356	−1.760	0.0002	NP_001092284	SRL	sarcalumenin	2.00E-05
wu:fb15e04	ENSDART00000124926	chr21:24464941-24474823	−2.510	0.0003	NP_476429	KRT3	keratin 3	5.00E-77
zgc:112964	NM_001013342	chr16:2281217-2297724	−3.510	0.0000	NP_001073906	SNED1	sushi,nidogen and EGF-like domains 1	3.00E-28
si:ch211-243g18.2	NM_001044910	chr10:9640875-9661489	−3.640	0.0000	NP_000413	KRT17	keratin 17	2.00E-72

Genes associated with the maturation of visual function are candidates for inherited human blindness. Indeed, in this study several genes previously linked to human retinal disease show significant differential expression during maturation of visual function. For example the human orthologs of pantothenate kinase 2 (*pank2*), retinal outer segment membrane protein 1 (*rom1*), phosphodiesterase 6A (*pde6a*), guanylate cyclase 3 (*gc3*) and retinitis pigmentosa 2 (*rp2*) genes are all associated with degenerative eye disease in humans and are up-regulated from 3–5 dpf in zebrafish eyes [37]–[40]. Genes encoding collagen, *col11a1* and *col2a1a*, are down-regulated from 3–5 dpf. The human orthologs of *col11a1* and *col2a1a* are associated with Stickler and Marshall syndromes, which cause visual dysfunction [41], [42]. These findings support the likelihood that other human orthologs of genes up-regulated in 3–5 dpf eyes may link with human retinal disease. Therefore, we determined which orthologs of the differentially expressed genes mapped to regions of the human genome linked with inherited retinal disease, but for which the causative gene remains unknown. Table 3 gives details of 40 inherited human retinal diseases and the genes associated with visual maturation that map near the disease locus.

**Table 3:** Zebrafish genes differentially expressed between 5 and 3 dpf were associated with human retinal diseases without molecular basis.

Diseases	OMIM ID	Chromosmal location	Homologous zebrafish genes differentially expressed between 5 and 3 dpf
Retinitis pigmentosa with mental retardation		Xp21-q21	*pim2*, *tfe3a*, *rpa2*, *dt1p1a10l*,*mao*, *ndnl2*, *snx12*, *wdr45*, *zc4h2*, *arr3l*, *rp2*,*atrx*, *tspan7*, *hdac8*, *rps6kal*, *pl10*, *pqbp1l*,*sypb*, *slc9a7*, *slc7a3*, *pja2*, *arhgef9*, *prickle3*
Dominant macular dystrophy, North Carolina type; dominant progressive bifocal chorioretinal atrophy	136550	6q14-q16.2	*ttk*,*syncripl*, *tmem30a*, *elovl4b*, *hmgn3*
Dominant macular dystrophy, cystoid	153880	7p21-p15	*igf2bp3*, *hdac9b*, *agr2*, *rpa3*, *arl4a*, *arl4ab*, *tmem106ba*, *nfe2l3*,*npy*, *tmem106b*, *macc1*, *cbx3b*
Dominant retinal-cone dystrophy 1	180020	6q25-q26	*sod2*, *ppil4*, *lrp11*
Dominant neovascular inflammatory vitreoretinopathy	193235	11q13	*wnt11r*, *capn1*, *actn3b*, *ucp1*, *npas4*, *drap1*, *pola2*, *yif1a*,*pygmb*, *hsp47*, *dpp3*, *kat5*, *gpr137*, *fkbp2*, *spcs2*, *rbm14*, *prdx5*, *dgat2*,*ctsf*, *cd248*,*chka*, *rnf121*, *peli3*
Recessive Joubert syndrome; recessive MORM syndrome	213300	9q34.3	*tubb2c*, *lcn15*, *agpat2*, *col5a1*, *man1b1*
Recessive nonsyndromal congenital retinal nonattachment	221900	10q21	*cdk1*
Recessive optic atrophy	258500	8q21-q22	*matn4*, *rpl7*, *cpne3*, *plekhf2*, *slc25a32a*, *ca2*, *wwp1*, *rims2*, *laptm4b*, *znf706*, *stmn2a*, *si:ch211-160k22.1*
X-linked progressive cone dystrophy 2	300085	Xq27	*fgf13*, *ube2n*
X-linked retinitis pigmentosa	300155	Xq26-q27	*fgf13*, *mmgt1*, *tfdp1*, *ube2n*
X-linked retinitis pigmentosa	300424	Xp22	*tmsb*, *pvalb9*, *rbb4l*, *sat1*, *egfl6*, *cnksr2*
X-linked retinitis pigmentosa	300605	Xq28-qter	*brd2a*, *cd99l2*,*bgn*, *ssr4*, *gdi1*, *g6pd*
X-linked optic atrophy	311050	Xp11.4-p11.2	*pim2*, *tfe3a*, *dt1p1a10l*,*mao*, *ndnl2*, *wdr45*, *rp2*, *tspan7*, *pl10*, *pqbp1l*,*sypb*, *slc9a7*, *prickle3*
Cone-rod dystrophy; de grouchy syndrome	600624	18q21.1-q21.3	*rx1*, *mbd2*,*lipg*, *eef2l2*, *nfe2l3*, *lman1*,*nars*,*mbd1*
Dominant macular dystrophy, North Carolina type; dominant progressive bifocal chorioretinal atrophy	600790	6q14-q16.2	*ttk*,*syncripl*, *tmem30a*, *elovl4b*, *hmgn3*
Recessive Refsum disease, adult form	600964	10p13	*gad2*, *epb4.1l4*, *vim*, *mpp7*, *myo3a*, *st8sia6*, *fam107b*, *gtpbp4*, *sephs1*, *rbm17*, *atp5c1*, *pfkfb3*, *arhgap12*, *akr1b15*, *dhtkd1*, *hspa14*, *sdhaf2*, *arhgap12a*,*pfkl*
Recessive Usher syndrome, type 1	602097	21q21	*zgc:110006*
Recessive retinitis pigmentosa	602594	16p12.3-p12.1	*plk1*, *chp2*, *zgc:153595*, *loc560874*, *gprc5b*
Recessive retardation, spasticity and retinal degeneration	602685	15q24	*neo1*,*phb*,*nptna*, *stra6*, *tspan3a*, *si:dkey-105e17.1*, *hmg20a*, *sin3a*, *neo1*
Dominant optic atrophy,Kjer type	605293	18q12.2-q12.3	*slc14a2*
Recessive cone-rod dystrophy	605549	1q23.1-q23.3	*celf3*, *hsp70l*, *mcl1a*, *atp1b1a*, *atp1b1b*, *ssr2*, *mcl1b*, *sf3b4*,*myoc*, *tpm3*, *syt11a*, *aldh9a1a*, *rab13*, *rgs5a*,*ubin*, *anp32e*, *selenbp1*, *ilf2*, *prrx1b*, *gpa33*, *pfdn2*, *tmco1*, *ufc1*, *cks1b*, *f11r*,*fdps*, *setdb1a*, *rbm8a*, *psmd4b*, *psmb4*,*ctsk*, *ctssb.2*, *wbp2*, *pygo2*, *si:ch211-284a13.1*,*udu*, *krtcap2*, *clk2*, *si:ch211-184m19.1*, *mllt11*, *pbxip1a*
Dominant familial exudative vitreoretinopathy	605750	11p13-p12	*fabp10a*,*ppib*, *caprin1a*, *hipk3*
Recessive retinitis pigmentosa	606068	2p15	*tmsb*, *meis1*, *cyp26b1*, *pno1*, *rab1a*, *suclg1*, *slc1a4*, *cnrip1*,*snrpg*, *acta1a*, *dusp11*, *atoh8*, *egr4*, *fabp1a*, *ccdc142*, *anxa4*
Recessive Senior-Loken syndrome; recessive nephronophthisis, Adolescent	606995	3q22.1	*bfsp2*
Recessive Joubert syndrome	608091	11p12-q13.3	*cry3*,*mdka*, *fabp10a*, *capn1*, *zp2.3*, *actn3b*, *ypel3*, *fen1*, *hsd17b12b*, *npas4*, *slc43a1a*, *drap1*, *pola2*, *yif1a*, *rom1*, *atg13*,*pygmb*, *tcn2*, *psmc3*, *dpp3*, *kat5*, *gpr137*, *fkbp2*, *ms4a17a.11*, *rbm14*, *prdx5*, *ddb2*,*ctsf*, *sdhaf2*, *cd248*,*incenp*,*chka*,*madd*, *dhx9*, *pla2g15*, *mapk8ip1*, *peli3*
Recessive Leber congenital amaurosis	608553	1p36	*ctnnbip1*, *sepn1*,*pgd*, *cdc42*, *rer1*, *ela3l*, *ela2*,*hnrnpc*,*nudc*,*tardbp*,*srm*,*ddost*, *padi2*, *eno1*, *atad3b*, *rcc2*, *zgc:136474*, *angptl7*, *casp9*, *tpx2*, *gale*, *sst3*, *plod1a*, *errfi1*, *igsf21b*, *rap1gap*, *plekhg5*, *hp1bp3*,*gabrd*, *kiaa0090*, *mfn2*, *e2f2*, *lactbl1*
Dominant macular dystrophy, late onset; dominant macular dystrophy with lens zonules;	608752	11q23.3	*arcn1*, *hyou1*, *bace1*, *sc5dl*, *apoa1*, *usp2a*,*mll*
Dominant macular dystrophy	608850	5p15.33-p13.1	*sdha*,*aktip*, *trip13*, *dnajc21*, *prdm9*, *enoph1*, *tars*, *sub1*, *c9*, *skp2*,*rictor*, *sb:cb734*, *ube2ql1*, *march6*
Recessive retinitis pigmentosa with posterior column ataxia (PCARP)	609033	1q32.3	*btg2*, *prox1*, *ptgs2a*,*aspm*, *atf3*, *camk1g*,*pdca*, *camk1g*, *b3galt2*, *nucks1*, *klhl12*, *smyd2a*, *eef1a1*,*mybph*, *pkp1*
Recessive foveal hypoplasia and anterior segment dysgenesis	609218	16q23.2-q24.2	*cotl1*, *osgin1*, *zc3h18*, *jph3*
Recessive retinitis pigmentosa, severe	609913	1p21.2-p13.3	*psma5*, *abcd3a*, *hiat1b*, *amy2a*, *prmt6*, *dennd2d*, *col11a1*, *cnn3b*, *rnpc3*,*mybph*,*agl*, *ptbp2*, *slc6a17*, *ntng1*
Dominant retinitis pigmentosa	610359	2q11.2	*zgc:66433*, *dusp2*, *lonrf2*
Dominant optic atrophy	610708	22q12.1-q13.1	*maff*, *pvalb2*, *mcm5*,*selm*, *drg1*,*mb*, *ewsr1b*, *myh9*, *slc16a8*, *sgsm3*, *pik3ip1*, *ewsr1a*, *tcn2*, *kdelr3*, *rnf185*, *csnk1db*, *zgc:76871*, *rbfox2*, *tomm22*, *apol1*, *cacng2*, *mkl1*
Dominant cavitary optic disc anomalies	611543	12q13.13-q14.3	*copz1*, *cry1a*, *cry1b*,*npffl*, *mdm2*, *krt4*,*dcn*, *mkrn1*, *scn8aa*, *hsp90b1*, *dusp6*,*pah*, *zgc:64098*, *nr4a1*, *ppp1r12a*,*snrpfl*,*pmelb*, *tuba1l*, *ube2n*, *chpt1*,*ung*, *cdk2*, *tmed2*, *ptpn11b*, *krt18*, *btg1*, *dhrs9*, *atp5g2*, *dazap2*, *ctdsp2*, *tuba1l2*, *ckap4*, *arpc3*, *msrb3*, *slc9a7*, *cela1*, *slc38a4*, *tbc1d15*,*iscu*, *col2a1a*, *ric8b*,*rarga*,*ctdspla*, *znf385a*, *anks1b*, *atp2a2b*, *stat2*, *acss3*,*hnrnpm*, *csrnp2*
Dominant macular dystrophy, benign concentric annular		6p12.3-q16	*ttk*,*syncripl*, *tmem30a*, *fbxo9*, *tram2*, *bmp5*, *elovl4b*, *rcan2*, *ptp4a1*, *hmgn3*, *lmbrd1*, *eef1a1*,*mut*
Cone-rod dystrophy		17q	*spon2b*, *ppm1e*, *bactin1*, *actc1a*, *cmlc1*, *psme3*, *nme2b.2*, *unc119b*, *kcnh6*,*aldocb*, *col1a1a*,*col1a1b*, *hoxb3a*, *traf4b*, *kpna2*, *fzd2*, *gngt2a*, *birc5a*, *sepw2b*, *sepw2a*, *aanat1*, *ddx5*, *znf207b*, *tob1b*, *psmd12*, *cx43.4*, *smarce1*, *eftud2*, *sec14l1*, *slc9a3r1*, *klhl11*, *fkbp10*, *pdk2*, *srsf2*, *psmb3*, *socs3a*, *psmd11b*, *wipi1*, *msi2b*,*phb*, *srsf1a*, *tob1a*, *usp36*, *atp5g2*, *arl4d*, *arl5c*, *csnk1db*, *tmem49*, *mettl3*, *nptx1*, *med24*, *krt17*, *suz12a*, *psmc5*,*prkca*, *gdpd1*, *ccdc47*, *osbpl7*, *grb2*, *prkar1aa*, *mettl2a*, *krt17*, *mrpl12*, *rgs9*, *wbp2*, *nmt1a*, *zgc:112372*,*thraa*, *krt1-19d*, *ca10a*, *mmd*, *acsf2*, *zgc:153240*, *cuedc1*, *krt20*, *abi3*,*hlf*, *psmd11a*, *taf15*, *akap1b*, *nbr1*, *abca5*,*acaca*, *msl1*, *cq108_danre*, *spata20*, *p4hb*, *leprel4*, *loc560874*, *nbr1*
Dominant macular dystrophy, North Carolina-like with progressive sensorineural hearing loss		14q11.2	*psmb5*,*vmhc*, *smyhc1*,*hnrnpc*, *mettl3*,*vmhcl*, *abhd4*,*homez*, *dad1*,*homez*,*nars*
Dominant macular dystrophy		19q13.31-q13.32	*ckma*,*apoeb*,*ckmb*, *calm3a*, *gps2*, *sae1*,*apoe*, *bbc3*,*relb*
Recessive retinitis pigmentosa		4q32-q34	*hand2*,*glrba*, *spcs3*, *hmgb2l*, *gucy1a3*, *spock3*, *fbxo8*
Recessive vitreoretinal dystrophy		22q13	*maff*, *pane1*, *samm50*, *slc16a8*, *sgsm3*, *rangap1*, *kdelr3*, *csnk1db*, *tomm22*, *mapk11*,*tef*,*tspo*, *sult4a1*,*selo*, *saps2*, *mkl1*, *fam118b*, *frmpd1*
Recessive retinitis pigmentosa		2p23.3	*dpysl5a*, *smarce1*, *krtcap3*,*hadha*,*hadha*, *uts1*, *si:dkey-34f16.5*, *adcy3*

### Gene Ontology analysis of genes differentially expressed during maturation of visual function

**Figure 2:**
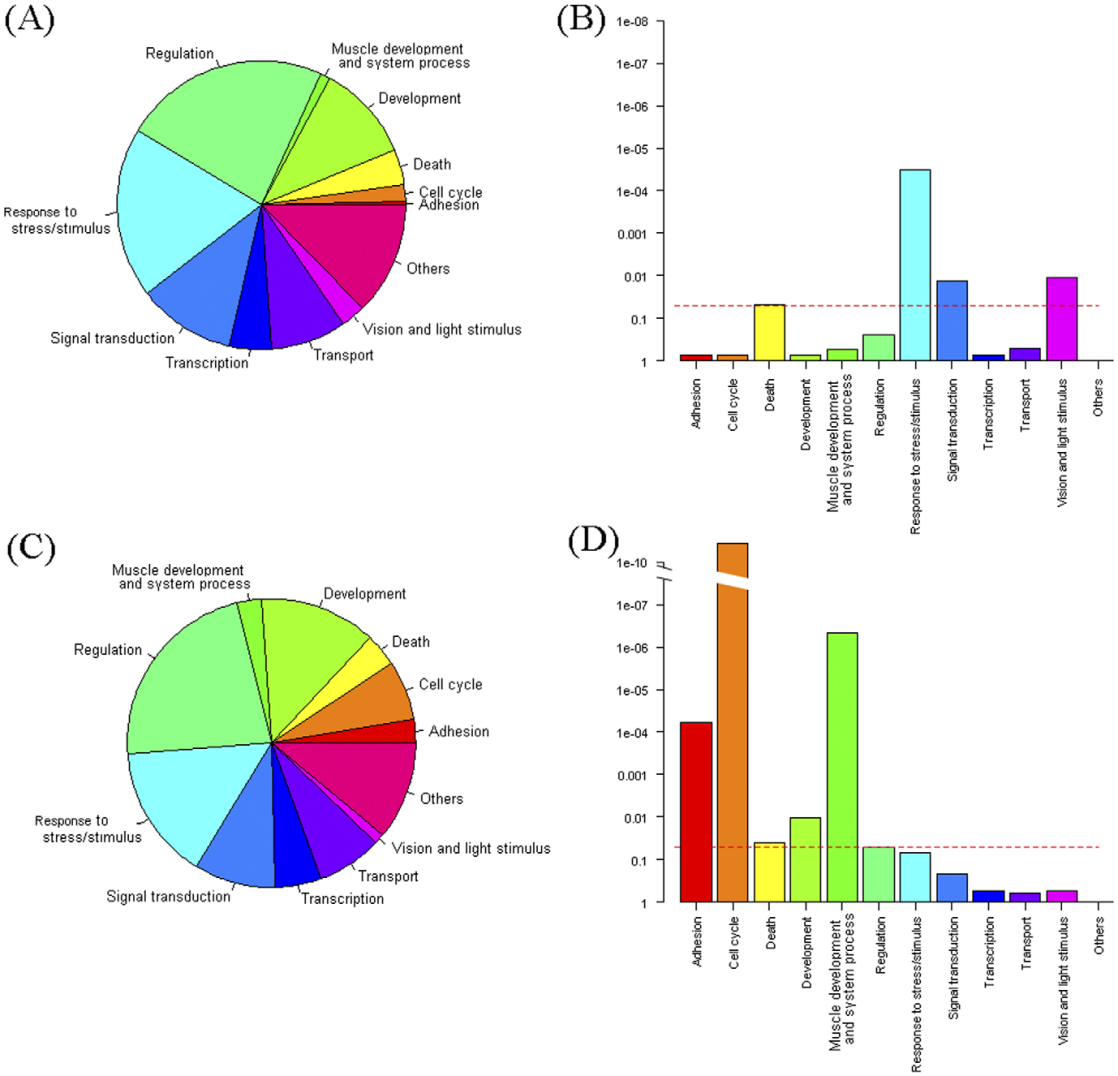
Summary of differentially expressed genes between 5 versus 3 dpf eyes using GO biological process annotation. A, B: GO summary for up-regulated genes between 5 versus 3 dpf. C, D: GO summary for down-regulated genes between 5 versus 3 dpf. A, C: pie chart presenting the number of differentially expressed genes in each GO category. B, D: bar plot presenting the significance of each GO term in Q-values. Q-value<0.05 was set as the significance threshold as depicted by the red dashed line.

We next sought to identify biological pathways enriched during development of visual function using Gene Ontology (GO) [19] and KEGG pathway [18] analysis. To enhance the functional annotation of our dataset, the human GO and pathway annotations were combined with the zebrafish annotation and Fisher's exact test was applied to select significantly enriched gene sets. For a global view of the biological processes associated with maturation of visual function, the differentially expressed genes were classified into standard GO terms (Figure 2). 5 dpf eyes show distinct enrichment of genes associated with “response to stress/stimulus”, “signal transduction” and “vision/light stimulus”. 3 dpf eyes are enriched for genes linked with “adhesion”, “cell cycle”, “development”, and “muscle development”.

Table 4 documents the more specific GO terms within Biological Process, Cellular Component and Molecular Function that exhibit significant enrichment during visual development. For gene sets enriched in 5 dpf eyes, the term “response to light stimulus” was significantly enriched, as expected. Interestingly, “response to cytokine stimulus” and “type I interferon-mediated signaling pathway” were high ranking terms in genes up-regulated in 5 dpf eyes. Enrichment of the term “response to cytokine stimulus” was interesting as this mode of cell signalling, typically via the Jak-Stat pathway, regulates diverse cells functions [9].

**Table 4:** Top 3 over represented GO terms by the differentially expressed genes between 5 versus 3 dpf for each GO category.

GO term	No. of differentially expressed genes in the GO term	No. of all genes annotated with the GO term	Q-value
**GO terms enriched with up regulated genes between 5 versus 3 dpf**			
**Biological Process**			
GO:0050896 response to stimulus	22	62	0.0014
GO:0060337 type I interferon-mediated signaling pathway	9	13	0.0014
GO:0034097 response to cytokine stimulus	11	19	0.0014
GO:0009416 response to light stimulus	12	23	0.0016
**Cellular Component**			
GO:0005576 extracellular region	54	285	0.0547
GO:0005741 mitochondrial outer membrane	13	43	0.0680
GO:0005740 mitochondrial envelope	6	12	0.0680
GO:0030133 transport vesicle	5	10	0.0993
**Molecular Function**			
GO:0003913 DNA photolyase activity	7	9	0.0028
GO:0046983 protein dimerization activity	14	41	0.0279
GO:0008236 serine-type peptidase activity	8	18	0.0536
GO:0004114 3,5-cyclic-nucleotide phosphodiesterase activity	4	5	0.0547
**GO terms enriched with down regulated genes between 5 versus 3 dpf**			
**Biological Process**			
GO:0000278 mitotic cell cycle	61	159	0.0000
GO:0000082 G1/S transition of mitotic cell cycle	44	94	0.0000
GO:0031145 anaphase-promoting complex-dependent proteasomal ubiquitin-dependent protein catabolic process	35	65	0.0000
GO:0000216 M/G1 transition of mitotic cell cycle	33	62	0.0000
**Cellular Component**			
GO:0000502 proteasome complex	28	48	0.0000
GO:0005783 endoplasmic reticulum	91	343	0.0000
GO:0005788 endoplasmic reticulum lumen	22	43	0.0000
GO:0005581 collagen	10	11	0.0000
**Molecular Function**			
GO:0008307 structural constituent of muscle	12	18	0.0000
GO:0005201 extracellular matrix structural constituent	11	16	0.0000
GO:0005509 calcium ion binding	50	191	0.0000
GO:0000166 nucleotide binding	145	783	0.0002

### Enrichment of Jak-Stat pathway genes during maturation of visual function

**Figure 3:**
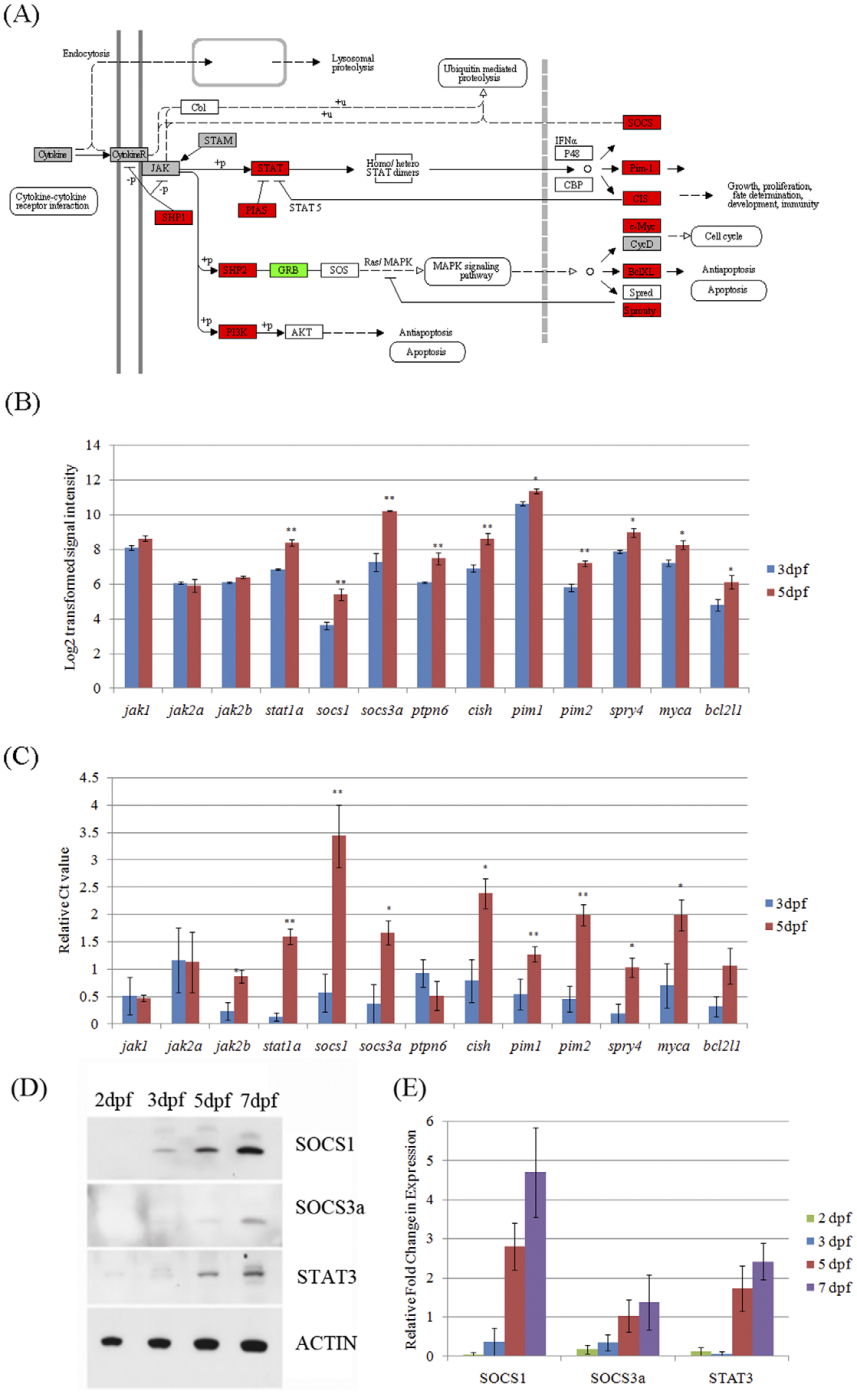
Up-regulation of gene and protein expression in the Jak-Stat signaling pathway from 3 to 5 dpf during eye development. (A) Red blocks are up-regulated genes between 5 versus 3 dpf. Green blocks are down-regulated genes between 5 versus 3 dpf. Grey blocks are genes not changed between 5 versus 3 dpf. White blocks are genes not targeted by the microarray or not in the zebrafish genome. The gene interaction network figure is from the KEGG database with modification adapted to zebrafish genes. (B) Signal intensities on the microarrays. *: q-value<0.05. **: q-value<0.01. (C) Real-time PCR results are depicted as relative abundance compared to lowest abundance sample. *: p-value<0.05. **: p-value<0.01. (D) Western blot and (E) densitometric analysis of protein samples extracted from zebrafish whole larvae shows that Socs1, Socs3a and Stat3 expression are up-regulated from 2 to 7 dpf.

KEGG, a literature-based pathways database, was used to profile pathways that were significantly enriched in genes associated with development of visual function [18] (Table 5). In 5 dpf eyes, the Jak-Stat and insulin signaling pathways were enriched in the up-regulated gene cohort (Figure 3A). This result is consistent with the GO analysis. Though the Jak-Stat pathway is known to regulate various developmental processes [43], [44], the role of this pathway during maturation of visual function in vertebrates is not well characterized. Thus, we sought to validate the enhanced expression of Jak-Stat pathway genes as visual function develops.

**Table 5:** Significantly over represented KEGG pathway by the differentially expressed genes between 5 versus 3 dpf.

KEGG Pathway	No. of differentially expressed genes in the pathway	No. of all genes annotated with the pathway	Q-value
**Pathways enriched with up regulated genes between 5 versus 3 dpf**			
Jak-Stat signaling pathway	13	29	0.0012
Insulin signaling pathway	21	70	0.0021
Pancreatic secretion	14	39	0.0036
MAPK signaling pathway	25	98	0.0038
Arginine and proline metabolism	12	32	0.0044
**Pathways enriched with down regulated genes between 5 versus 3 dpf**			
Proteasome	26	39	0.0000
Dilated cardiomyopathy	13	22	0.0003
ECM-receptor interaction	12	21	0.0007
Hypertrophic cardiomyopathy (HCM)	13	25	0.0009
Protein processing in endoplasmic reticulum	34	112	0.0012

Quantitative real-time PCR (qRT-PCR) of the expression of Jak-Stat signaling genes (e.g. *stat1a*, *socs1*, *socs3a*, *pim1*, *pim2*) are generally in good agreement with the microarray results with respect to the direction of signal changes and statistical significance (Figure 3B,C). Although, *jak2* and *bcl2l1* only demonstrate significant up-regulation by one method, these genes changed in the same direction and with similar amplitude using both methods. Only in the case of *ptpn6* were the microarray and QRT-PCR data contradictory. Immunoblot analysis was conducted on 2 to 7 dpf larvae to determine if the transcript changes observed were matched by changes in protein levels of Socs1 and Soc3a. Though Stat3 was not targeted by the microarray chip, it was also analysed because of its known regulator role in Jak-Stat signaling. All three proteins are detected at very low levels at 2 dpf (Figure 3D–E). However, Socs1, Socs3a and Stat3 did exhibit ∼100, ∼8 and ∼19 fold increases in expression from 2 to 7 dpf, consistent with the observed mRNAs increases.

### Developmental Expression Pattern of Jak-Stat Genes in zebrafish Eyes

**Figure 4:**
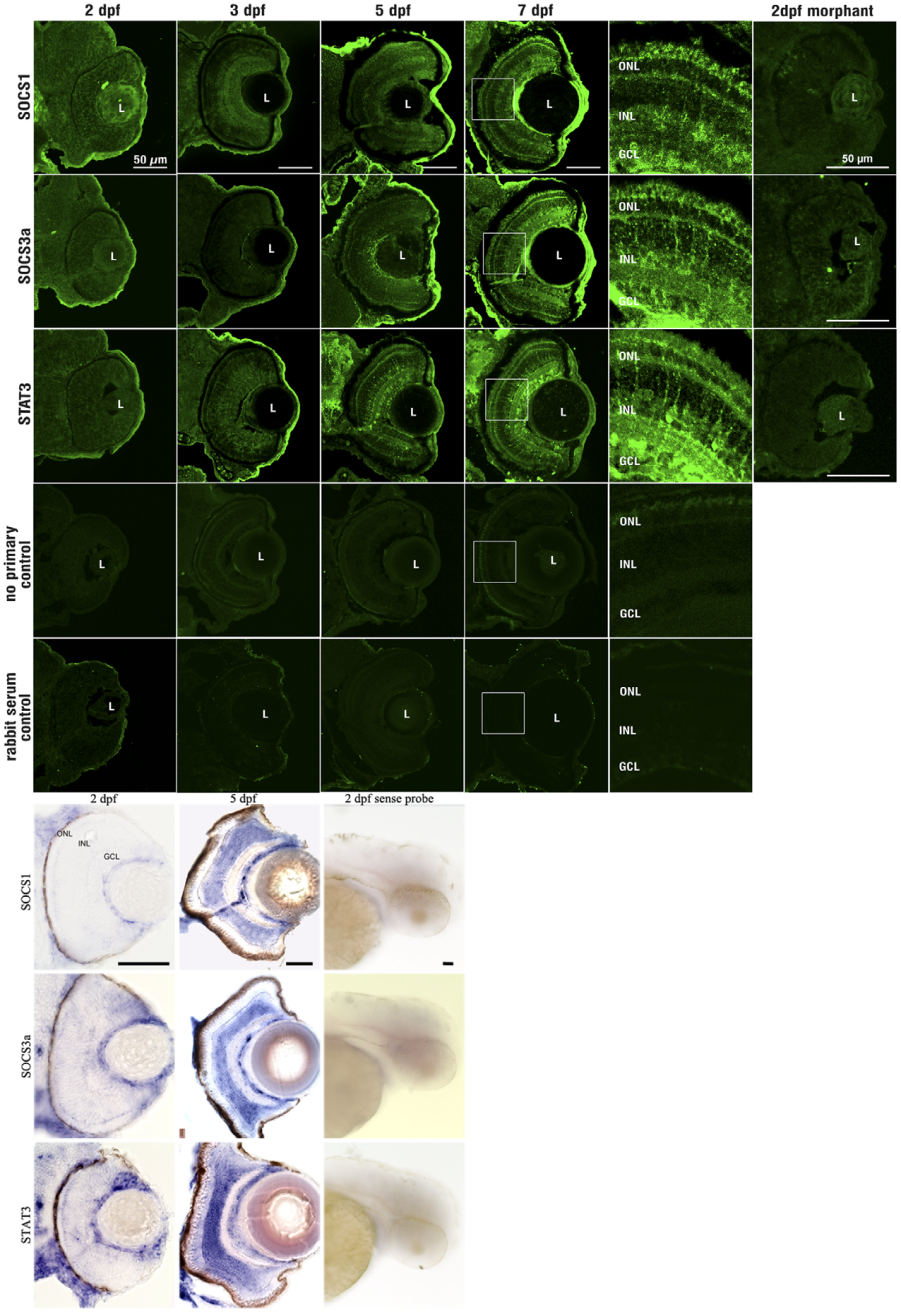
Socs3a, Socs1 and Stat3 expression in 2–7 dpf retina. First five panels are immunohistochemical analysis showing increasing expression of Socs3a, Socs1 and Stat3 throughout the retina from 2–7 dpf. Minimal staining is observed in secondary antibody alone, pre-immune serum or morphant controls. The last three panels are in-situ hybridisations of *socs3a*, *socs1* and *stat3* genes on 2 and 7 dpf. No significant staining was observed in sense probe controls. GCL, ganglion cell layer; INL, inner nuclear layer; ONL, outer nuclear layer.

Next we sought to determine the spatial expression patterns of Socs1, Socs3a and Stat3 in the maturing retina using *in situ* hybridization and immunohistochemistry (Figure 4A–B). At 2 dpf, *socs1, socs3a* and *stat3* RNAs appear to be expressed in the ganglion cell layer (GCL). By 7 dpf, *socs1*, *socs3a*, and *stat3* RNAs are expressed in the GCL and the inner nuclear layer (INL), additionally, *stat3* RNA appears to be expressed at low levels in the outer nuclear layer (ONL) (Figure 4B). By immunohistochemistry, Socs1, Socs3a, and Stat3 polyclonal antisera exhibit increasing expression in the neuroretina from 2 to 7 dpf (Figure 4A). At 7 dpf, the three proteins are detected throughout the neuroretina from the photoreceptor layer to the GCL.

**Figure 5:**
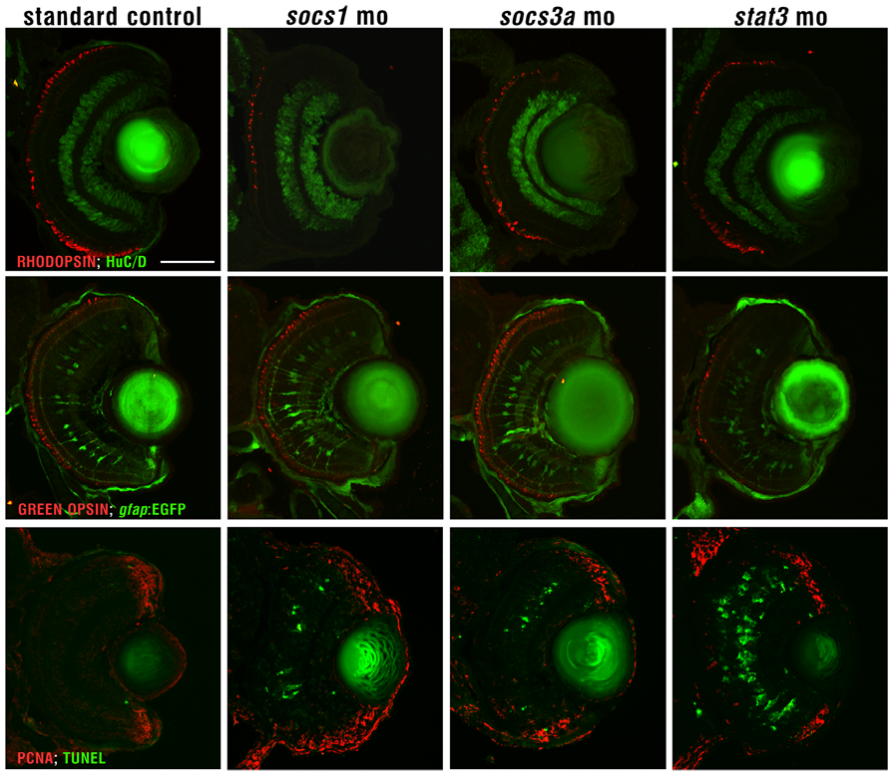
Cell types in Socs1, Socs3a and Stat3 morphant retinae. Socs1, Socs3a and Stat3 morphant retinae were labelled with rhodopsin for photoreceptors, gfap:GFP for Muller glia, Hu for inner retinal neurons and TUNEL staining for apoptotic cells. Scale bar in the upper left panel represents 50 microns and is the same for all the panels.

The expression of Socs1, Socs3a, and Stat3 suggests that these three proteins may play a role in retinal development. To examine this hypothesis, we knocked down the expression of each of the three proteins individually using morpholinos. The *socs3a* and *socs1* morphant retinae reveal no significant changes in the patterning of rod and cone photoreceptors (rhodopsin and green opsin, respectively), Muller glia (*gfap:GFP*) or inner retinal neurons (Hu). In contrast, the *stat3* morphants exhibit reduced numbers of cone photoreceptors (green opsin) and Muller glia. All three morphants also exhibit a small eye relative to the standard control morphant (Figure 5), which is likely due to the increased numbers of TUNEL-positive cells in the morphants relative to the standard control morphants.

### Pim kinases

**Figure 6:**
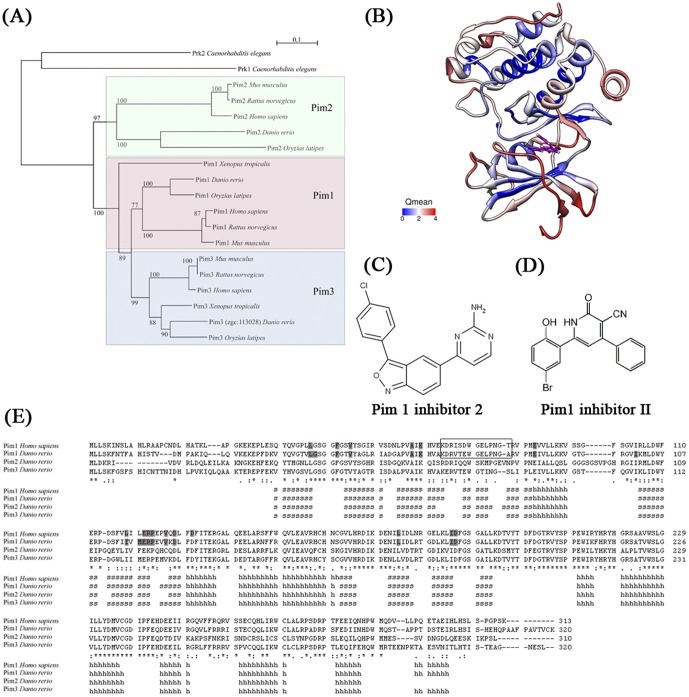
Sequence and structure analysis of Pim protein family. (A) Neighbor-joining tree with 1000 bootstrap resamplings. Subgroups for Pim1, Pim2 and Pim3 proteins are highlighted in different colors. *C. elegans* Prk proteins were used to root the tree. (B) The 3-D structure of zebrafish Pim1 protein was predicted by homology modeling using Swiss-Model [29] using the human PIM1 crystal structure 3BGP as the template. Estimated accuracy in Qmean value was colored from blue to red. A lower Qmean value indicates more reliable prediction. The Pim1 inhibitor 2 drug docking site was predicted using SwissDock [32]. (C) Chemical structure of Pim1 inhibitor 2. (D) Chemical structure of Pim1 inhibitor II. (E) Sequence alignment of zebrafish Pim proteins with human PIM1 with assigned secondary structure. Residues within 4.5 Å of the Pim1 inhibitor 2 docking site are highlighted in grey in human and zebrafish Pim1.The Pim1 antibody-binding site is highlighted using a rectangle.

Another Jak-Stat pathway gene whose enhanced expression in the eye correlates with maturation of visual function is *pim1*, and it became the focus of subsequent analyses. Socs3 is a negative regulator of Stat3 [45], Pim1 regulates the stability of Socs1 [46] and is a target of Stat3 [47] compounding our interest in *pim1*. Moreover, human PIM1 is an oncogene, thus an association with visual function was intriguing. Pim1 is a serine-threonine kinase, known to suppress apoptosis and promote cell cycle progression [48]–[50]. In humans, the PIM kinase gene family includes three functionally redundant paralogs, PIM1–3. In zebrafish, *pim1* and *pim2* were previously annotated. Due to high sequence similarity with its human homolog, we identified *zgc:113028,* a novel zebrafish gene, as a *pim3* ortholog in zebrafish. Phylogenetic analyses demonstrate that Pim kinases are highly conserved in vertebrates and share similar evolutionarily conserved positions (Figure 6A). The zebrafish Pim1 kinase has a high degree of sequence identity (73%) with human PIM1, which suggests a common 3-D structure. Thus, we constructed a 3-D model of zebrafish Pim1 kinase from the published crystal structure of human PIM1 [29] (Figure 6B). Interestingly, the inner pocket of the ATP-binding domain was predicted with high accuracy, indicating structural conservation of zebrafish and human Pim1 proteins. In silico drug docking analyses also predict that Pim1 inhibitor 2 can dock in the ATP-binding domain of zebrafish Pim1 (Figure 6 B, E). Although not definitive, these analyses provide support that PIM1 antibodies and inhibitors can also target zebrafish Pim1.

### Ocular expression of Pim1 kinase

**Figure 7:**
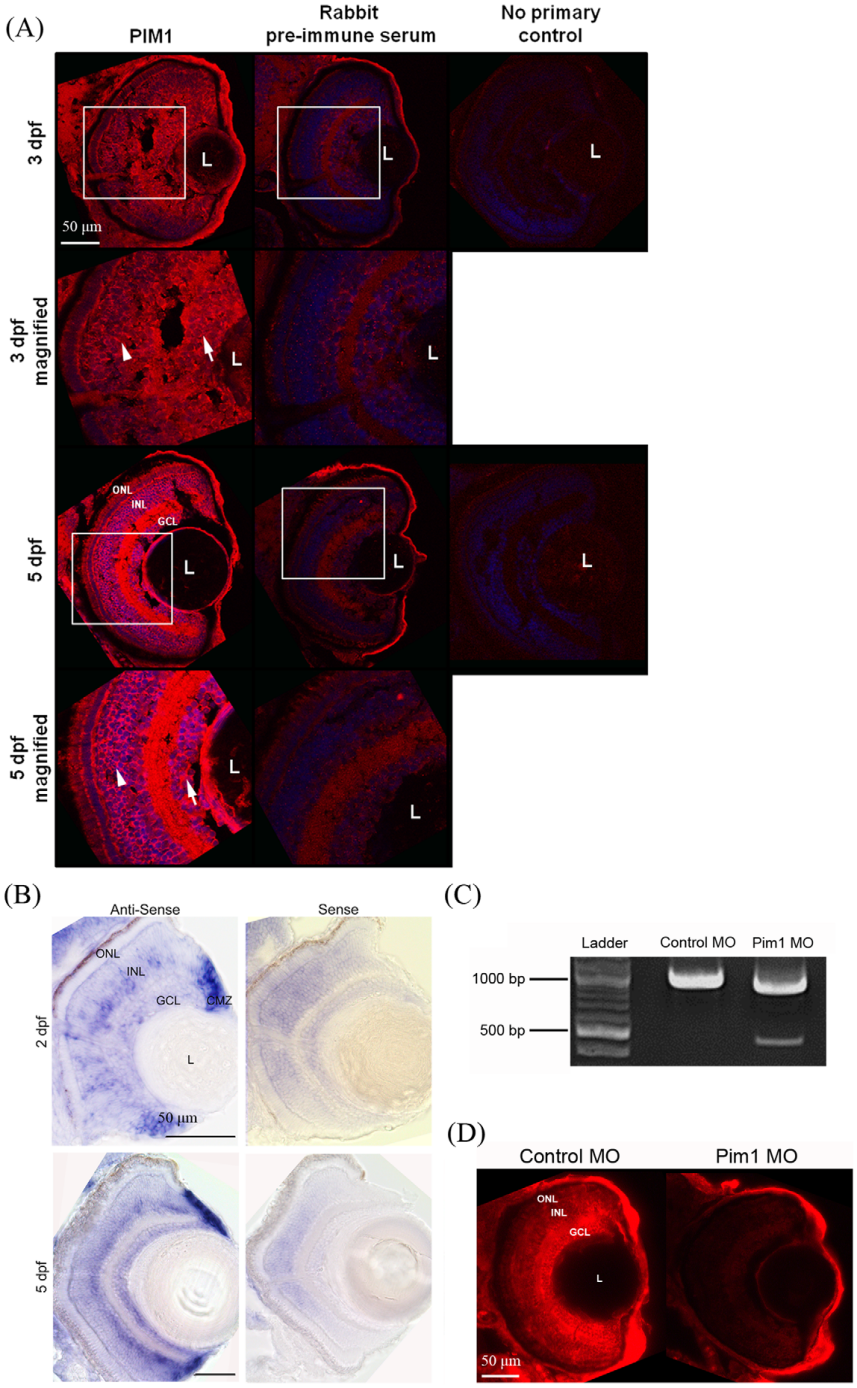
Localisation of Pim1 in the larval eye. (A) Immunohistochemical analysis of Pim1 protein (red) and DAPI (blue) in 3 and 5 dpf larval eyes reveals Pim1 expression throughout the neuoretina including the ganglion cell layer (arrow) and inner nuclear layer (arrowhead) at both timepoints. (B) Pim1 RNA is expressed in the INL, GCL and CMZ of 2 and 5 dpf larvae. (C) RT-PCR amplification of *pim1* from 5 dpf cDNA results in a 1059 bp band in control morpholino-injected larvae and a second ∼500 bp band in *pim1* splice site morpholino-injected larvae consistent with knockdown of *pim1*.(D) Pim1 expression is present in the ONL, INL and GCL of 5 dpf larvae injected with 0.025 mM standard control morpholino but highly reduced in 5 dpf larvae injected with 0.025 mM pim1 morpholino. White boxes indicate the areas magnified. MO, morpholino; ONL, outer nuclear layer; INL, inner nuclear layer; GCL, ganglion cell layer; CMZ, ciliary marginal zone; L, lens.

An antibody targeting K71toT84 of human PIM1, a region which has 11 of 14 amino acids conserved with zebrafish Pim1, was used for immunohistochemistry on zebrafish retinal sections (Figure 7A). At 3 dpf, the Pim1 antibody detects low expression levels in the neuroretina. Expression observed in the lens and cornea was considered non-specific, as it is also observed with pre-immune serum. At 5 dpf, stronger, specific staining with the Pim1 antibody is observed throughout the neuroretina. By in situ hybridization, *pim1* exhibits increased expression in the GCL and INL layers from 2 to 5 dpf (Figure 7B). *Pim1* was also expressed strongly in the ciliary marginal zone (Figure 7B). Microinjection into zebrafish embryos of *pim1* morpholinos that disrupt splicing of *pim1* (Figure 7C) results in a specific diminishment of the staining of Pim1 in the retina (Figure 7D). The expression of *pim1* RNA and Pim1 protein are reminiscent of the staining observed with the other Jak-Stat signaling proteins Socs1, Socs3a, and Stat3, and consistent with the microarray and qRT-PCR. Overall, these results indicate enhanced expression of Pim1 throughout the zebrafish neuroretina from 3–5 dpf..

### Inhibition of Pim kinase specifically suppresses visual function in zebrafish larvae

**Figure 8:**
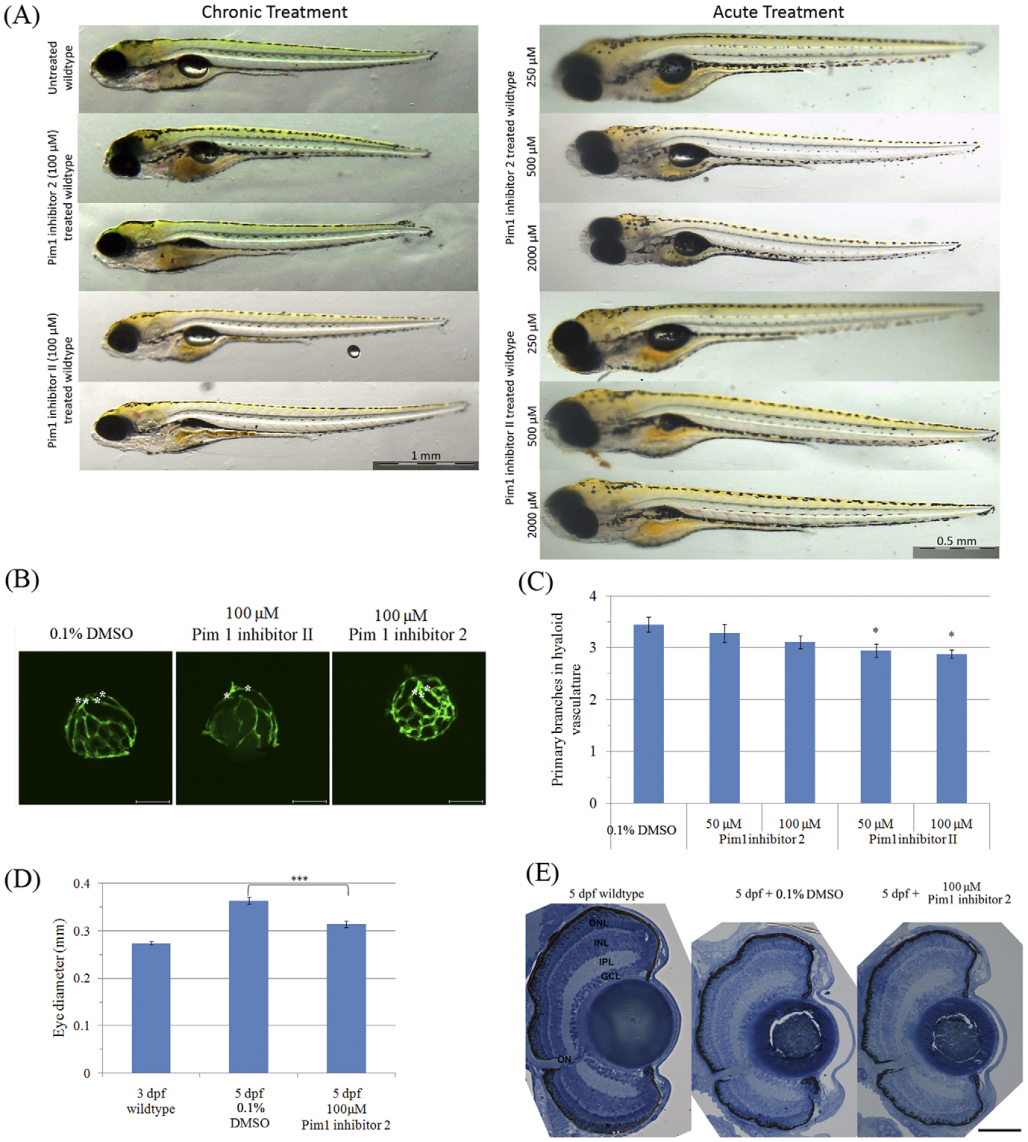
Larvae treated with Pim1 inhibitors have a slightly reduced number of primary hyaloid vessels and eye size. (A) Representative images of whole zebrafish larvae treated using Pim1 inhibitors. (B) and (C) zebrafish (n = 17 to 20) treated with Pim1 inhibitor 2 from 3–5 dpf have normal hyaloid vasculature morphology, while treatment with Pim1 inhibitor II from 3–5 dpf slightly reduces the number of primary hyaloid vessels. Primary hyaloid vessels are pointed using asterisks. P-value was calculated using one way ANOVA with Dunnett's correction for multiple comparisons. *:ANOVA p<0.05. (D) Zebrafish larvae treated with Pim1 Inhibitor 2 from 3–5 dpf have a smaller eye. ***: Student's t test p<0.001. (E) Retinal lamination appears normal in the larvae with drug-treated from 3–5 dpf. Scale bars are 1 mm (A) and 50 mm (E).

**Figure 9:**
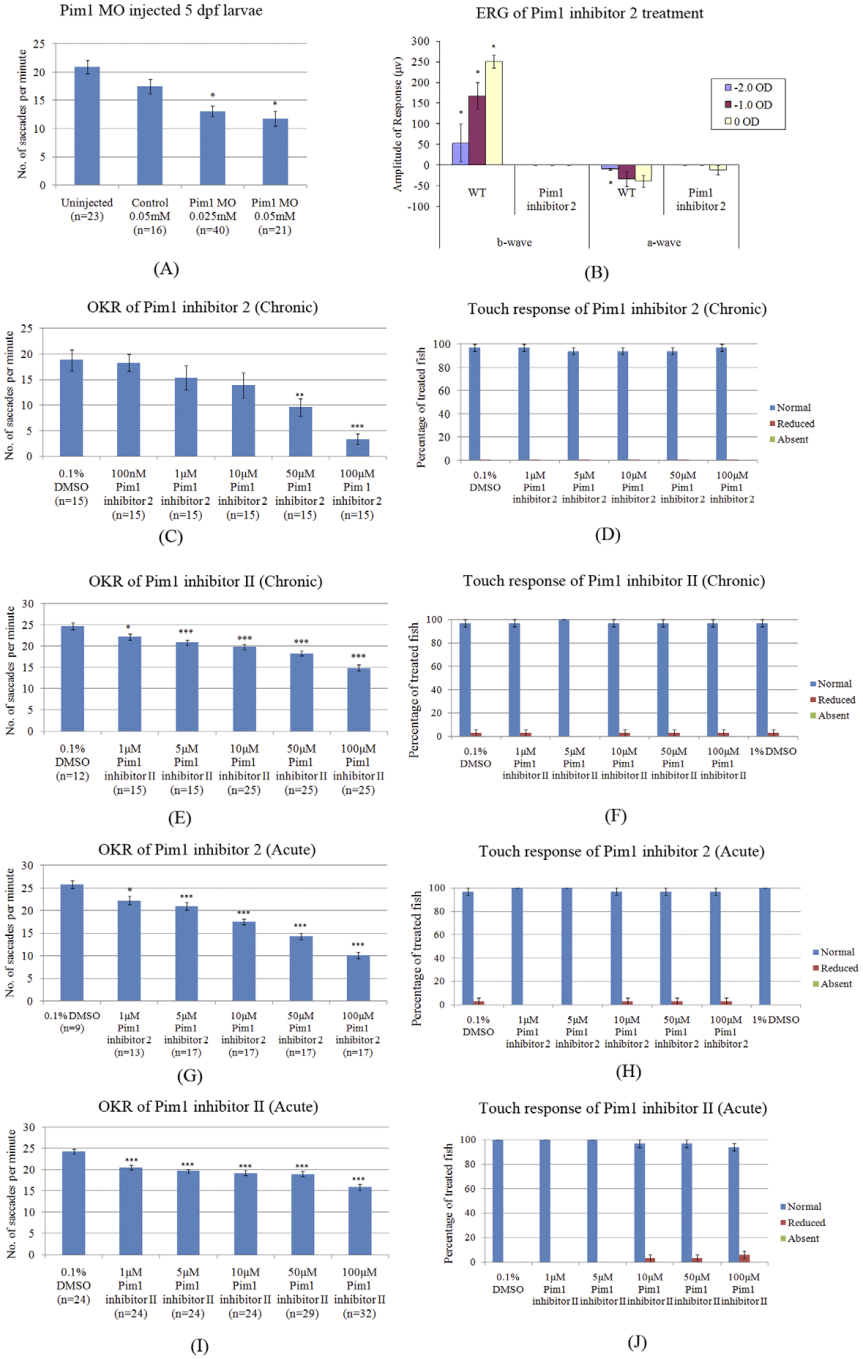
Pim1 inhibition results in a reduced visual response at 5 dpf. (A) The OKR of Pim1 MO injected fish is significantly reduced. Larvae were treated with increasing concentrations of Pim1 inhibitor 2 or Pim1 inhibitor II ranging from 1 to 100 µM. Pim1 inhibitors were dissolved in 0.1% DMSO at all concentrations except the 100 µM concentration which was dissolved in 1% DMSO. (B) The ERG a-wave and b-wave are significantly decreased in 100 µM Pim1 inhibitor 2 treated fish. (C–J), significant reduction of OKR saccades in zebrafish treated from 3–5 dpf (“chronic”) or treated for 1 hour at 5 dpf (“acute”) using Pim1 inhibitors. The locomotor touch response of fish treated with Pim1 inhibitors is unaltered. p-values were calculated using Student's t test. *: p<0.05. **: p<0.01. ***: p<0.001.

To investigate the role of Pim1 in the retina, we performed loss of function experiments. Initially, we treated larvae from 3–5 dpf (“chronic”) with two different Pim1 inhibitors (Figure 6 C,D). No gross morphological defects were observed aside from occasional un-inflated swim bladders at the highest concentrations, a phenotype not observed with subsequent “acute” inhibitor treatments for 1 hour only at 5 dpf (Figure 8A). The histology of the eye was also largely unaffected (Figure 8E) except for a slight reduction in eye diameter and in the average number of primary branches of hyaloid vasculature (Figure 8C–D). We also determined that concentrations of Pim1 inhibitor 2 or II up to 2000 µM did not cause lethality or gross morphological defects in zebrafish (Figure 8A) and that concentration up to 100 µM had no effect on the touch response, a locomotor response independent of vision (Figure 9). These experiments indicate that the Pim1 inhibitors are well tolerated by the larvae and that they do not induce significant morphological or general locomotor defects.

**Figure 10:**
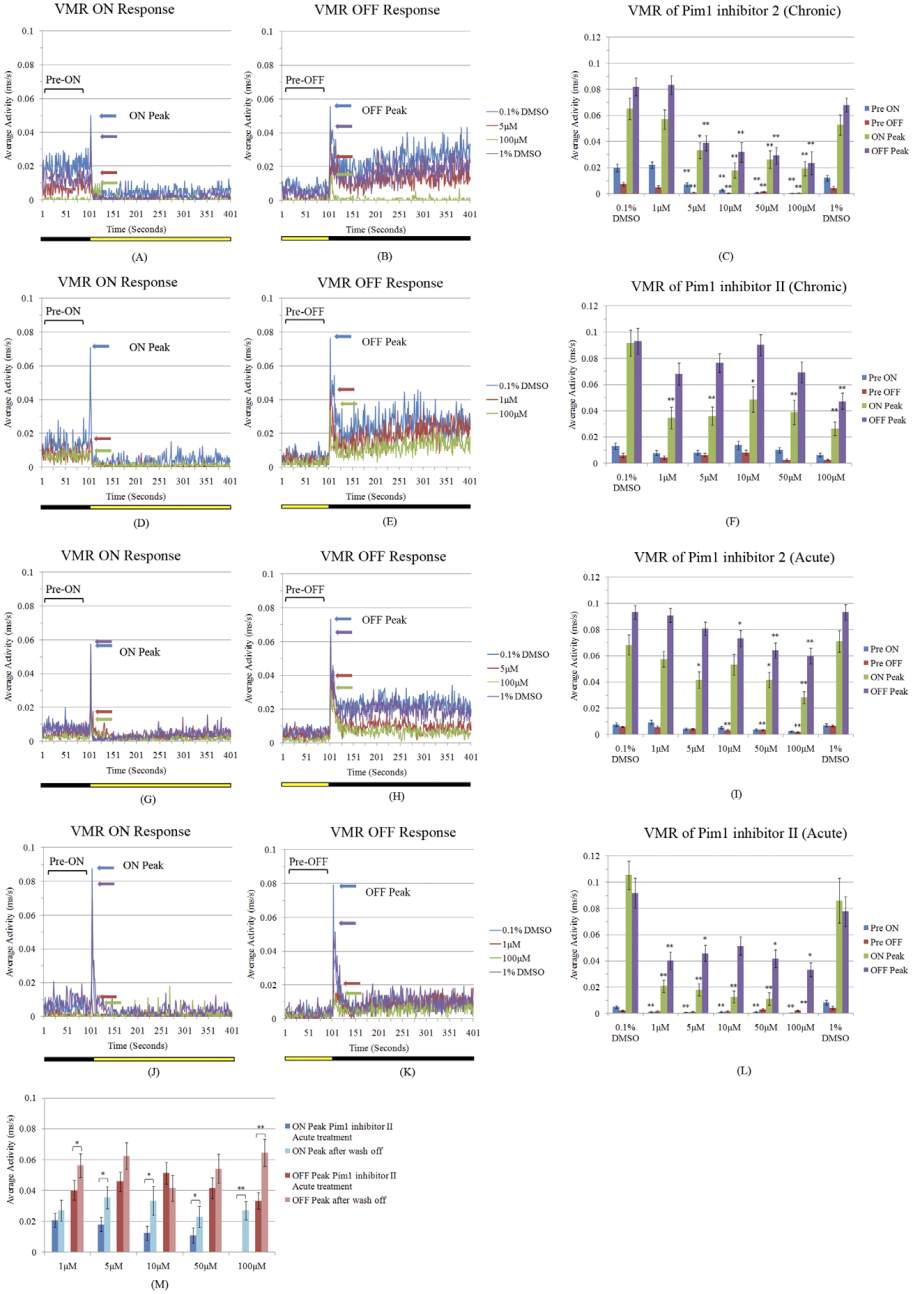
Pim1 inhibition results in reversible reduction of VMR on and off peaks. Zebrafish (n = 36 to 60) were treated from 3–5 dpf (“chronic”) or at 5 dpf for 1 hour (“acute”) using Pim1 inhibitors. (A–L) The ON and OFF response are significantly reduced in zebrafish treated using Pim1 inhibitors compared to zebrafish treated with DMSO control. p-values were calculated using Wilcoxon rank sum test with Bonferroni correction for multiple testing. *: p<0.05, **: p<0.01. (M) After 1 hour Pim1 inhibitor II treatment at 5 dpf, zebrafish larvae were washed with embryo medium and settled for 7–8 hours before the VMR was re-tested. After removal of Pim1 inhibitor II, zebrafish showed significantly recovered ON and OFF responses. p-values were calculated using Wilcoxon rank sum test. *: p<0.1. **: p<0.01.

The role of Pim1 in visual function was then investigated by assessing loss-of-function effects on the OKR. Knockdown of Pim1 using splice-site blocking morpholinos injected at embryonic stages results in a significant reduction in the number of saccades at 5 dpf, compared to control morpholino injected larvae (Figure 9A). In agreement, “chronic” treatment of larvae with Pim1 inhibitors from 3–5 dpf also results in significant, dose-dependent diminishment of the number of OKR saccades and of the peak VMR responses to light changes (Figure 9 and 10). As these assays do not exclusively analyse ocular function, we quantified outer retinal function to different light flash intensities by ERG (Figure 9B). The ERG a-wave is produced by photoreceptors and the b-wave represents neurotransmission from light-activated photoreceptors to bipolar cells. Larvae treated with Pim1 inhibitor 2 from 3–5 dpf exhibited a-wave and b-wave amplitudes reduced by ∼2–4 fold, with the larger reductions at higher light intensities.

To determine if “acute” treatment with Pim1 inhibitors could alter visual function, 5 dpf larvae were treated for only 1 hour before analysis of the OKR and VMR (Figure 9 and 10). Again, significant diminishment of the OKR and the VMR peak responses were observed with Pim1 inhibitor 2 and Pim1 inhibitor II at concentrations that *i)* are substantially lower than the maximum tolerated concentration, *ii)* which do not affect the touch locomotor response and *iii)* that do not affect retinal morphology. In order to ensure the inhibitor induced reductions on VMR peaks are vision driven events, VMR on and off peaks were normalized to pre-on and pre-off activities, respectively. The normalized peaks represent fold increases in locomotor activity after light change. Notably, significant reductions in normalized on and off peaks were observed with Pim1 inhibitors treatments (Figure S1).

Moreover, reversibility of drug action was confirmed in zebrafish acutely treated with Pim 1 inhibitor II for 1 hour at 5 dpf (Figure 10M). Treated zebrafish were washed with embryo medium and the VMR tested after 7–8 hours settling. Peak on and off light responses after drug removal showed significant improvement from Pim1 inhibitor-treated fish indicating that acute effects on visual behaviour were not due to drug toxicity. In summary, perturbation of Pim1 kinase results in specific diminishment of visual function.

## Discussion

Genes differentially expressed in 3–5 dpf zebrafish eyes were profiled to identify potential novel regulators of visual function maturation. Interestingly, genes comprising the Jak-Stat signalling pathway were found to be most enriched from 3 to 5 dpf. Janus kinase (JAK) is a key regulator of interferon and cytokine signalling [9]. Receptor binding results in downstream activation of signal transducer and activator of transcription (STAT) factors, which regulates target gene transcription in the nucleus. This study focussed on a downstream target of the Jak-Stat pathway, the Pim1 oncogene, as its role in visual function had not previously been appreciated. Pim genes encode serine threonine kinases, which are important downstream effectors in cytokine signalling [10]. They have been shown to play a role in promoting cell proliferation and in inhibiting apoptosis [51]. However, our study suggests a novel role for Pim1 in visual function, independent of these processes.

In *Drosophila*, the Jak-Stat pathway regulates various developmental processes including embryogenesis, hematopoiesis, organ development and sex determination [43]. The Jak homolog Hop and the Stat homolog STAT92E are known to mediate *Drosophila* eye imaginal cell growth and differentiation [52], [53]. SOCS36E, dPIAS and dBRWD3, regulators of Jak-Stat signalling, are also essential in determining *Drosophila* eye size and visual function [54]. Moreover, the Jak-Stat pathway interplays with Hh, mTOR and Notch pathways to form a gene regulatory network for *Drosophila* eye development [54]. In vertebrates, Jak-Stat signalling is more complicated due to complex signalling inputs, gene redundancy and networking [44]. In the eye, ciliary neurotrophic factor (CNTF) is a potent cytokine that activates Jak-Stat to regulate vertebrate eye development [55], [56]. CNTF binding to its receptor gp130 activates JAK protein kinases (Jak1, Jak2 and Tyk2) and subsequent phosphorylation of latent transcription factors STAT1 and STAT3. During mouse embryonic eye development, Jak2, Tyk2, STAT1 and STAT3 exhibit strong expression in the developing ganglion cell layer and inner plexiform layer [57]. Later at postnatal stages, these components are localized to the ganglion cell layer, the inner nuclear layer, and the two plexiform layers. Other Jak-Stat components are also known to regulate eye development. SOCS3, the negative feedback modulator of STAT3, is required for rhodopsin expression and rod photoreceptor cell differentiation [45]. SOCS3a is required for optic nerve regeneration [58]. While there is evidence that components of the Jak-Stat pathway are expressed and play various important roles in the developing eye, the expression and function of many other Jak-Stat pathway genes in visual development is largely unknown.

Here, we quantify visual behavior responses and confirm that zebrafish show significant maturation of visual function between 2 and 5 dpf. This gain of visual function appears independent of gross morphological changes to the eye, as the patterned retina, lens and cornea are already present by 3 dpf. Microarray and qRT-PCR demonstrate that many Jak-Stat genes are significantly enriched in the vertebrate eye as visual function matures. This includes *jak*, *stat* and *socs*, and many downstream genes including *ptpn6*, *cish*, *pim1*, *pim2*, *spry4*, *myca* and *bcl21l1*. Because the Affymetrix GeneChip only represents a subset of zebrtafish genes, other Jak-Stat genes are expected to be differentially expressed during visual function development. For example, Stat3 is not targeted by the GeneChip probes, but at the protein level it exhibits higher ocular expression at 5 and 7 dpf (Figure 5). Furthermore, immunostaining confirms Socs1, Socs3a, Stat3 and Pim1 are expressed at low levels in the early developing retina but have stronger and broader expressed in the laminated retina. Stat3 and Socs3 have similar expression patterns in the embryonic zebrafish eye compared to mouse [45], [59]. This indicates an evolutionary conserved pattern of expression of the Jak-Stat signaling pathway during eye development.

It was intriguing that enhanced expression in the eye, of an ortholog of the PIM1 oncogene, correlated with gain of visual function. PIM kinases are associated with various human cancers, including prostate, oral, colon, pancreatic and lymphoma [60]–[63]. *Pim1*–3 gene paralogs encode serine threonine kinases, which are important downstream effectors in cytokine signalling [10]. STAT transcription factors can directly bind to *pim* promoter sequences and PIM kinases can negatively regulate the Jak-Stat pathway by binding to the negative regulator SOCS proteins [47], [64]–[66]. Although initially linked primarily with haematopoiesis, Eichmann *et al.* previously suggested novel functions of Pim kinases outside the haematopoietic system, particularly in epithelia and the CNS [10]. During early mouse development, *pim* genes have overlapping or complementary expression in the hematopoietic system, epithelia and central nervous system [10]. Of particular relevance to this study is the reported expression of *pim1* in the neural retina of embryonic mice [10]. However, an association of Pim proteins with visual function was not previously reported.

Overall, our data supports a novel role for Pim1 kinase in visual function. At stages post-retinogenesis, pan-retinal staining of Pim1 is enhanced in larval zebrafish as vision matures. In addition, Pim1 inhibitors or Pim1 knockdown results in diminished visual behaviour. The diminished OKR or VMR could result from defects in the eye, brain or musculature. However, the normal locomotor response to a tactile object indicates that non-visual locomotor responses and the musculature are unaffected, whereas the abnormal ERG indicates that the retina is affected. Defects in visual function could also arise from toxic effects to the fish or morphological abnormalities. However, the concentrations of Pim1 inhibitor that produce visual behaviour defects are at least 10–1000 fold lower than the maximum tolerated concentrations and no significant morphological defects were observed in the eye. When treated from 3–5 dpf, there is small effect of Pim inhibitors on primary hyaloid vessel branch number. This phenotype is unlikely to account for the defects in visual behaviour associated with Pim1 inhibition, because 1 hour treatment, which does not affect hyaloid branch number, can still reduce visual behaviour. In addition, other studies demonstrate that a reduced number of primary hyaloid vessels did not result in visual behaviour or retinal function defects [34]. Finally, removal of the Pim1 inhibitors results in almost fully restored visual behaviour after 8 hours, supporting a specific action of the drugs.

Our findings highlight the need for further research into the role of Pim1 in visual function in normal and diseased situations. We speculate that loss of Pim1 results in signalling defects in the retina that perturb visual function without affecting retinal morphology. Disruption of visual function in the retina does not have to occur by degenerative mechanisms and can result from deficits in chromophore levels, phototransduction or synaptic transmission. For example, a similar perturbation of visual function has previously been reported upon Jak-Stat activation in the retina following exogenous CNTF treatment [67]. Indeed other kinases are known to regulate visual function; rhodopsin kinase regulates phototransduction, phosphatidylinositol-3-kinase-like kinase (PIKK), cAMP-dependent protein kinase, and the tyrosine kinase insulin receptor regulate photoreceptor synaptic transmission and cAMP-dependent protein, PKC, CaM Kinase, MAP kinase and src family kinases modulate synaptic exocytosis [68]–[70]. Targets of Pim1 kinase include transcription regulators and proteins involving in cell cycle progression and apoptosis [71]. Two Pim1 targets, Socs1 [66] and Myca [72], are shown in this study to be up-regulated from 3 to 5 dpf during vision function maturation (Figure 3). However the mechanism of disturbance of visual function by Pim1 inhibition still needs further investigation. In summary, we uncover correlations between expression levels of Jak-Stat pathway genes with maturation of visual function, and demonstrate an unforeseen role of the Pim1 kinase in visual function.

## Supporting Information

Table S1 **List of primers for real-time PCR and in situ hybridization.**
(XLSX)

Figure S1 **Pim1 inhibition results in reduction of normalized VMR on and off peaks.**
(PDF)
