# Supplementary material for: Inhibition of the Pim1 Oncogene Results in Diminished Visual Function
Source: PLoS One. 2012 Dec 26;7(12):e52177. doi: 10.1371/journal.pone.0052177 (PMC3530609; doi:10.1371/journal.pone.0052177)
Supplement: Figure S1 — Pim1 inhibition results in reduction of normalized VMR on and off peaks. (PDF) [file pone.0052177.s002.pdf]

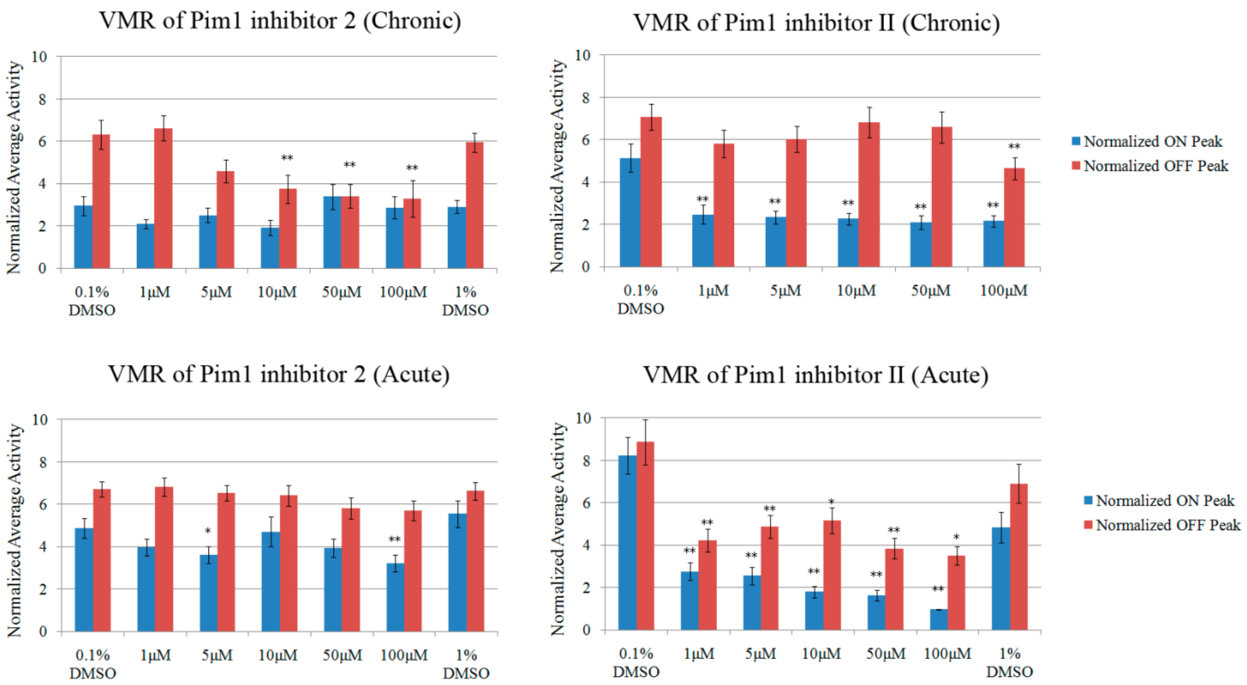

Supplementary Figure S1. Pim1 inhibition results in reduction of normalized VMR on and off peaks. VMR on and off peaks were normalized by dividing pre-on and pre-off activities. Significant reductions were observed in the Pim1 inhibitors treatment. *, Wilcox rank sum p<0.05. **, Wilcox rank sum p<0.01.
